# Anti-Inflammatory Medicinal Plants of Bangladesh—A Pharmacological Evaluation

**DOI:** 10.3389/fphar.2022.809324

**Published:** 2022-03-24

**Authors:** Most. Afia Akhtar

**Affiliations:** Department of Pharmacy, Faculty of Science, University of Rajshahi, Rajshahi, Bangladesh

**Keywords:** Bangladeshi, traditional, inflammation, medicinal plants, preclinical study

## Abstract

Inflammatory diseases are considered major threats to human health worldwide. In Bangladesh, a number of medicinal plants have been used in traditional medicine from time immemorial in the treatment of diverse diseases, including inflammatory disorders. This assignment aims at providing the status of the medicinal plants of Bangladesh which are traditionally used in the management of inflammatory disorders and are investigated for their anti-inflammatory prospects using different preclinical studies and future research directions. The information of medicinal plants assembled in this review was obtained from a literature search of electronic databases such as Google Scholar, PubMed, Scopus, Web of Science and ScienceDirect up to December, 2020 from publications on plants investigated for their anti-inflammatory activities, in which the place of plant sample collection was identified as Bangladesh. Keywords for primary searches were “anti-inflammatory,” “Bangladeshi,” and “medicinal plants.” Criteria followed to include plant species were plants that showed significant anti-inflammatory activities in 1) two or more sets of experiments in a single report, 2) same or different sets of experiments in two or more reports, and, 3) plants which are traditionally used in the treatment of inflammation and inflammatory disorders. In this study, 48 species of medicinal plants have been reviewed which have been used in traditional healing practices to manage inflammatory disorders in Bangladesh. The mechanistic pathways of the *in vivo* and *in vitro* study models used for the evaluation of anti-inflammatory properties of plant samples have been discussed. Selected plants were described in further detail for their habitat, anti-inflammatory studies conducted in countries other than Bangladesh, and anti-inflammatory active constituents isolated from these plants if any. Medicinal plants of Bangladesh have immense significance for anti-inflammatory activity and have potential to contribute toward the discovery and development of novel therapeutic approaches to combat diseases associated with inflammation. However, the plants reviewed in this article had chiefly undergone preliminary screening and require substantial investigations including identification of active molecules, understanding the mechanism of action, and evaluation for safety and efficacy to be followed by the formulation of safe and effective drug products.

## Introduction

Plants are the most abundant suppliers of safe and successful remedies from time immemorial to present either to humans or to other animals. It is estimated that more than 90% of traditional medicine recipes comprise medicinal plants (Sofowora et al., 2013) which are used to treat a wide array of acute and chronic diseases ranging from common cold to complex cancerous phases throughout the world (Issa et al., 2006). According to the World Health Organization, the use of traditional and complementary medicine is increasing rapidly in most of the countries (World Health Organization, 2013). Medicinal plants constitute 25% of all modern medicines (Birhanu, 2013), and the annual market value of these plants has surpassed $100 billion globally (Sofowora et al., 2013).

Plants are the reservoir of important bioactive molecules classified as phenolics, alkaloids, carotenoids, organosulfur compounds, etc. on the basis of their chemical nature, and these molecules are reclassified as antioxidants, analgesics, cardioactive, anticancerous, immunity potentiating, detoxifying, neuropharmacological agents, etc. on the basis of their pharmacological action (Ugboko et al., 2020). Thus, plants with values in traditional medicine integrated with scientific evidences have provided the opportunity to discover thousands of therapeutically potential drugs (Harvey, 2000).

### Traditional Background of Bangladeshi Medicinal Plants

Bangladesh is a small country regarding her land area, but the fertile soil and favorable climate have enriched the country with highly biodiverse plants. The traditional healing practices of Bangladesh have been exercised from time immemorial and are profoundly embedded within the local communities (Ocvirk et al., 2013; Haque et al., 2018). A myriad of medicinal plants grows all over Bangladesh out of which about 1,000 are conservatively considered to have therapeutic usefulness by traditional healers (Mollik et al., 2010). The formulations of Ayurvedic, Unani, and Homeopathic systems of this country have been developed by exploring these natural resources (Rahman et al., 2001; Ghani, 2003). Even at present, traditional medicine is an integral part of the country’s overall healthcare system (Haque et al., 2014; Jahan et al., 2019). The usage of medicinal plants in the form of extract, decoction, juice, powder, paste, etc. in such traditional practices possesses the same long history to manage or cure diverse diseases (Akber et al., 2011; Rahmatullah et al., 2011a). The major reasons people of Bangladesh rely on these medicinal plants are 1) little or no access to modern medical assistance, 2) availability and cost-effectiveness of medicinal plants, and, 3) trust in the healing power of these natural gifts that has been built up with time, observations, and experiences. In addition, more than 30 tribes constitute 2% of the total population of Bangladesh, and the tribal healers are their principal health care providers who again rely on medicinal plants for treatment of different diseases (Rahmatullah et al., 2011b; 2012).

The increase in prescription rate and popularity of herbal medicine indicates the shift of the global trend from synthetic drugs toward the medicines of natural origin which has also been considered a promising future medicine (Ahmad Khan and Ahmad, 2019). Similarly, in Bangladesh, the manufacturing of herbal medicines has been increased, and the demand for medicinal plants is also increasing (Dulla and Jahan, 2017). But these plants are yet to undergo detailed scientific investigations for chemical constituents and bioactivities to evaluate their pharmacological properties (Rahman et al., 2001), for instance, anti-inflammatory potential.

### Inflammation, Inflammatory Disorders, and Available Anti-Inflammatory Medication

The term “inflammation” is described as a prompt and strictly controlled physiological process (Barton, 2008) triggered by harmful foreign stimuli as well as infected and injured host tissue (Medzhitov, 2008; 2010). The foreign stimuli include pathogenic microbes, toxic chemicals, allergens, mechanical and thermal factors, etc. (Ambriz-Pérez et al., 2016; Arulselvan et al., 2016; Attiq et al., 2018). If not properly coordinated, this natural beneficial physiologic action persists instead of being resolved and evolves pathological consequences, leading to the development and progression of numerous human diseases which encompass asthma, rheumatoid arthritis, inflammatory bowel disease, cancer, atherosclerosis, type 2 diabetes, obesity, and neurodegenerative disorders, for example, Alzheimer’s disease, Parkinson’s disease, and multiple sclerosis (Nathan, 2002; Glass et al., 2010; Medzhitov, 2010; Fürst and Zündorf, 2014). These inflammatory diseases are the major health issues around the globe, causing an increase in the rate of morbidity and mortality every year (Gou et al., 2017).

Developing an efficacious anti-inflammatory drug product with a higher margin of safety has always been a challenge. The currently prescribed common anti-inflammatory drugs can be divided into three classes 1) nonselective non-steroidal anti-inflammatory drugs (NSAIDs), 2) cyclooxygenase 2 (COX-2) selective NSAIDs, and, 3) steroidal anti-inflammatory drugs (SAIDs) (Recio et al., 2012). NSAIDs act by retarding the biosynthesis of prostanoids from arachidonic acid (AA) by inhibiting cyclooxygenase (COX) enzymes. These COX enzymes can exist as the constitutive COX-1 and the inducible COX-2 isoforms. Though responsible to mediate inflammation, the ubiquitous COX-1 isoform mainly performs physiologic functions associated with homeostasis as well as protection of cells and tissues accompanying the endothelium, monocytes, gastrointestinal epithelial cells, and platelets. On the other hand, COX-2 is mostly induced by cytokines expressed in the vascular endothelium, rheumatoid synovial endothelial cells, monocytes, and macrophages and plays the key role in inducing pain and inflammation (Vane and Botting, 1998; Smyth et al., 2009; Brune and Patrignani, 2015). The nonselective NSAIDs inhibit both COX-1 and -2, and thus besides providing therapeutic actions, their use can result in a number of undesired side effects (Table 1) (Davies, 1995). The selective COX-2 inhibitors were considered to be therapeutically superior to the conventional nonselective NSAIDs since they have little or no effect on COX-1 isozymes (Everts et al., 2000; Jackson and Hawkey, 2000), but in later studies, these drugs have also been found to cause cardiovascular events such as myocardial infarction, stroke, and heart failure as well as gastrointestinal (GI) complications (Table 1) (Salvo et al., 2011). The other class, that is the steroids, is potent anti-inflammatory agents which can inhibit phospholipase A_2_ (PLA_2_) enzymes required to release AA from phospholipids. AA liberates eicosanoids, for instance, prostaglandins, thromboxanes, leukotriene, etc. and platelet-activating factor (PAF) which are the principal inflammatory mediators (Balsinde et al., 2002; Ericson-Neilsen and Kaye, 2014). Adverse reactions associated with high dose or long-term use of small dose of steroid tablets and with inhaled steroids are summarized in Table 1 along with the adverse reactions of NSAIDs and COX-2 selective NSAIDs.

**TABLE 1:** Cellular action mechanism of anti-inflammatory medications and their undesired effects.

Types of anti-inflammatory drugs	Cellular action mechanism in brief	Example	Undesired effects
Nonselective nonsteroidal anti-inflammatory drugs (NSAIDs)	Inhibit both COX-1 and -2 isozymes	Aspirin, acetaminophen, ibuprofen, naproxen etc.	Colonic bleeding, iron deficiency anemia, strictures, ulcerations, perforations, inflammatory bowel disease (IBD), diarrhea, hepatotoxicity, cardiovascular diseases, and death
COX-2 selective NSAIDs	Selectively inhibit COX-2 isozyme	Celecoxib, etoricoxib, and rofecoxib (rofecoxib has been withdrawn from the market due to its adverse effects)	Increased risk of heart attack, stroke, and GI events such as perforation, ulceration, and bleeding
Steroidal anti-inflammatory drugs (SAIDs)	Act *via* inhibiting PLA_2_ enzymes	Tablet form such as cortisol, prednisolone, prednisone, and methylprednisolone	Bruising of the skin, weight gain, osteoporosis, diabetes, cataracts, swelling of the ankles or feet, joint destruction, impaired wound healing, excessive hair growth, fat redistribution, atherosclerosis, and hypertension
		Inhaled form such as beclomethasone, budesonide, flunisolide, fluticasone propionate, and triamcinolone	Sore mouth, hoarse voice, and infections in the throat and mouth

Sources: (Salvo et al., 2011; Recio et al., 2012; Akhtar, 2018).

### Anti-Inflammatory Compounds of Plant Origin

**FIGURE 1:**
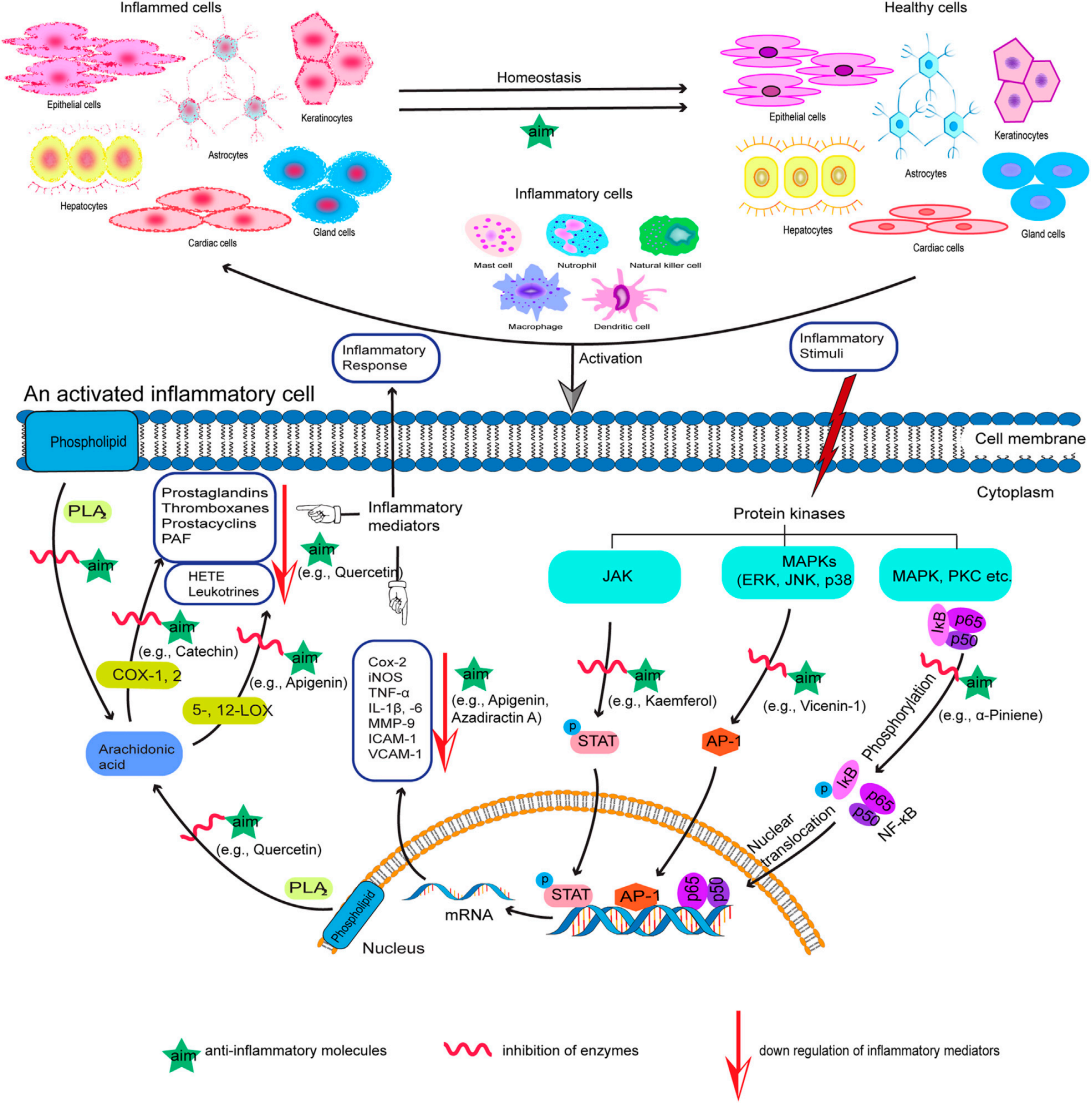
Proposed action mechanism of anti-inflammatory molecules (aim) of plant origin with examples. The specific pathways shown represent only a few of the plenty of diverse pathways involved in the inflammatory process.

In recent years, research interest on screening of medicinal plants to discover potential anti-inflammatory agents has been intensified (Bellik et al., 2012; 2013). A variety of plant-derived natural products have been shown to exhibit significant anti-inflammatory activity by inhibiting important proinflammatory mediators (Figure 1) and have made way from preclinical studies to clinical trial (Kim et al., 2009; Fürst and Zündorf, 2014). For example, quercetin and kaempferol inhibit inducible nitric oxide synthase (iNOS) expression and signal transducer and activator of transcription-1 (STAT-1) and nuclear factor-κB (NF-κB) activation (Hämäläinen et al., 2007); resveratrol inhibits COX-2 expression followed by modulation of NF-κB and activator protein-1 (AP-1) pathways (Kundu et al., 2006); curcumin, the principal curcuminoid of turmeric (*Curcuma longa* L., Zingiberaceae), acts *via* inhibiting COX-1 and -2, lipoxygenase (LOX), NF-κB, and mitogen-activated protein kinase (MAPK) and also downregulates tumor necrosis factor-α (TNF-α) and interleukin-1β and 6 (IL-1β, and -6) secretion (Hong et al., 2004; Kim et al., 2005; Shah et al., 2010). Table 2 summarizes some of the known anti-inflammatory molecules of plant origin.

**TABLE 2:** Major chemical classes of plant-derived anti-inflammatory molecules.

Secondary metabolite class	Examples	References
Alkaloids	Aconitine, berberine, colchicine, cepharanthine, capsaicin, ephedrine, and pseudo-ephedrine	Ivanovska and Philipov (1996); Barbosa-Filho et al. (2006); Souto et al. (2011)
Essential oils	β-myrcene, limonene, cinnamaldehyde, eugenol, anethole, caryophyllene, 1,8-cineole, and α-pinene	Santos and Rao (2000); Souza et al. (2003); Tung et al. (2008); Miguel (2010); de Cássia da Silveira et al. (2014); Kim et al. (2015b); Özbek and Yılmaz (2017)
Flavonoids	Apigenin, epigallocatechin gallate, genistein, hesperidin, kaempferol, luteolin, myricetin, quercetin, and rutin	Guardia et al. (2001); Kim et al. (2004); González-Gallego et al. (2007); Gomes et al. (2008); Tunon et al. (2009)
Glycosides	Digitoxin, digoxin, ouabain, codonolaside, and oleuropein	Miles et al. (2005); Xu et al. (2008); Fürst et al. (2017)
Phenolics	Lapachol, ellagic acid, caffeic acid, catechol, oleocanthal, bergenin, cannabichromene, tremetone, and vanillic acid	de Almeida et al. (1990); Perez (2001); Miles et al. (2005); Kassim et al. (2010); Kazłowska et al. (2010); Beg et al. (2011); Lucas et al. (2011)
Saponins	Fruticesaponin A-C, ruscogenin, capillarisin, kalopanaxsaponin-A, monodesmosides, ginsenosides, prosapogenin D methyl-ester, and buddlejasaponin I	Just et al. (1998); Huang et al. (2008); Attiq et al. (2018)
Stilbenes	Resveratrol, oxyresveratrol, desoxyrhapontigenin, aiphanol, and isorhapontigenin	Wadsworth and Koop (1999); Guardia et al. (2001); Fang et al. (2008); Choi et al. (2014)
Terpenoids	Artemisin, artemisinin, artemisolide, curcumin, zingiberene, helenalin, andrographolide, hispanolone, acanthoic acid, carnosol, carnosic acid, triptolide, kamebanin, oridonin, ursolic acid, oleanolic acid, betulinic acid, glycyrrhizin, β-carotene, lycopene, and lupeol	Fernández et al. (2001); Salminen et al. (2008); de las Heras and Hortelano (2009); Beg et al. (2011); Kashyap et al. (2016)

Apu et al. (2012) published a brief review on anti-inflammatory medicinal plants of Bangladesh in 2012, where they enlisted 36 plants. Another report briefly reviewed 15 analgesic and anti-inflammatory medicinal plants of Bangladesh in addition to cognitive enhancer plants (Uddin and Islam, 2020). In the present study, 48 plants have been chosen on the basis of some inclusion and exclusion criteria. These plants are reviewed for their traditional uses and anti-inflammatory activity in various *in vivo* and *in vitro* experimental models, and toxicity test results have also been summarized in Supplementary Table S1 if conducted in the cited research(s). More insights have been given into selected plants, highlighting their identified anti-inflammatory molecules. The compelling motive behind the current study is to represent the scope and possibilities of medicinal plants of Bangladesh with traditional values supported by scientific evidences in the treatment of inflammation and inflammatory disorders.

## Methods

The plant species included in this review (Supplementary Table S1) were based on the reports available from January, 2001 to December, 2020 by searching electronic databases such as Google Scholar, PubMed, Scopus, Web of Science, and ScienceDirect. Keywords used were primarily “anti-inflammatory,” “Bangladeshi,” and “medicinal plants.” A secondary search was conducted similarly with keywords “membrane stabilizing activity,” “Bangladeshi,” and “medicinal plants” since membrane stabilization is a well-documented mechanism of anti-inflammatory action, and the *in vitro* membrane stabilizing assay is established as a valid model to study the anti-inflammatory activity of extracts or molecules (Shinde et al., 1999; Thangaraj, 2016). In these published reports, the plants or their parts were collected from different areas of Bangladesh, and the experimental works were conducted in laboratories of Bangladesh and also in abroad as found in few studies.

The common name, local name, and traditional uses of plants included in Supplementary Table S1 were extracted mainly from the books of Professor Abdul Ghani and Sarder Nasir Uddin and from few other references. The database http://www.worldfloraonline.org/ (previously www.theplantlist.org) was followed for the accepted Latin name of each plant. Criteria followed to include plant species were plants that showed significant anti-inflammatory activities in 1) two or more sets of experiments in a single report, 2) same or different sets of experiments in two or more reports, and 3) plants which are traditionally used in the treatment of inflammation and inflammatory disorders. On the other hand, exclusion criteria include 1) reports not meeting the inclusion criteria, 2) plants reported with insufficient data or no data of doses of the sample or extract and/or positive control, and 3) plants showing anti-inflammatory activity in a single set of experiments in a single report. This review is, therefore, not exhaustive for all the medicinal plants of Bangladesh reported to have anti-inflammatory activity. The toxicity test results summarized in Supplementary Table S1 have been extracted from the same report reviewed for anti-inflammatory activity of plants if toxicity had been tested in the study.

## Result and Discussion

This study represents 48 medicinal plants of Bangladesh from 47 genera belonging to 29 families which have traditional values in the treatment of inflammatory disorders along with other medicinal uses. Inflammatory diseases such as arthritis, asthma, tumor, etc. have been managed conservatively using different parts or products of these plants (Supplementary Table S1). *Acanthus ilicifolius* L., *Acmella paniculata* (Wall. ex DC.) R.K.Jansen, *Aegiceras corniculatum* (L.) Blanco*, Ageratum conyzoides* (L.) L., *Alangium salviifolium* (L.f.) Wangerin, *Alocasia macrorrhizos* (L.) G.Don, *Argyreia argentea* (Roxb.) Sweet, *Azadirachta indica* A. Juss., *Cyanthillium cinereum* (L.) H.Rob., *C. patulum* (Dryand. ex Dryand.) H.Rob., *Glycosmis pentaphylla* (Retz.) DC*.*, *Heliotropium indicum* L., *Lantana camara* L., *Mangifera indica* L., *Manilkara zapota* (L.) P.Royen, *Mussaenda roxburghii* Hook.f., *Oroxylum indicum* (L.) Kurz, *Phrynium imbricatum* Roxb., *Phyllodium pulchellum* (L.) Desv., *Piper retrofractum* Vahl, *Steudnera colocasiifolia* K.Koch, *Swietenia mahagoni* (L.) Jacq., *Thunbergia grandiflora* (Roxb. ex Rottl.) Roxb., *Toona ciliata* M.Roem*.*, *Vigna unguiculata* (L.) Walp., *Vitex negundo* L., and *Withania somnifera* (L.) Dunal have values in the treatment of arthritis. *Clerodendrum infortunatum* L., *Coccinia grandis* (L.) Voigt, *Eclipta prostrata* (L.) L., *Euphorbia hirta* L., *Flemingia stricta* Roxb., *P. retrofractum*, and *Terminalia arjuna* (Roxb. ex DC.) Wight & Arn. have been used as a traditional remedy for asthma. Examples of plants credited for antitumor properties include *Butea monosperma* (Lam.) Taub., *Gynura nepalensis* DC., *Leea macrophylla* Roxb. ex Hornem., *Mallotus repandus* (Willd.) Müll.Arg., *Microcos paniculata* L., *Premna esculenta* Roxb., and *Typhonium trilobatum* (L.) Schott*.* Furthermore, *Aglaia cucullata* (Roxb.) Pellegr., *M. zapota*, and *Urena sinuata* L. are known to be efficacious against inflammation besides other traditional uses. These plants were reported to possess significant anti-inflammatory activities which were evaluated using various *in vivo* and *in vitro* experimental models. The mechanistic pathways of these models have been taken into consideration for discussion, which provides better understanding of the action mechanism of plants reviewed in this study.

### Common Experimental Methods to Evaluate Anti-Inflammatory Activity of Natural Products

Numerous biochemical mediators work jointly to commence and continue the inflammatory cascade. Crude extracts and/or pure compounds derived from plants target these mediators and have paved the way for the development of new therapeutic approaches. *In vitro* methods are mainly based on the inhibition of such activated mediators. The potent biochemical mediators include, enzymes (PLA_2_, COX-1, COX-2, 5-LOX, 12-LOX, 15-LOX, MMP-2, MMP-9, inosine monophosphate dehydrogenase, and β-hexosaminidase); free radicals (ROS, RNS, and SOD); prostaglandins (PGE_2_, TXA_2_; hydroxyleicosatetraenoic acid [HETE]); leukotrienes (LTB_4_, LTC_4_); cluster of differentiation molecules (CD-2, CD-11a, CD-11b, CD-18, and CD-49d), etc. (Venkatesha et al., 2011; Asante-Kwatia et al., 2020). Other important biochemical targets include proinflammatory cytokines (TNF-α, INF-γ, IL-1, IL-6, and IL-1β) and chemokines (IL-8, ICAM-1, and VCAM-1). In addition to these, a number of transcription factors including nuclear factor (NF)-κB, mitogen-activated protein kinases (MAPKs), extracellular signal–regulated kinase (ERK), c-Jun-N-terminal kinase (JNK), signal transducer and activator of transcription-1 (STAT-1), p38 kinases, and AP-1 have been used as molecular targets of inflammation (Luster et al., 2005; Venkatesha et al., 2011). Red blood cell (RBC) membrane stabilization and protein denaturation assays have also been reported in a vast number of studies.

On the other hand, commonly used *in vivo* methods are carrageenan-induced paw edema, croton oil or TPA (12-*O*-tetradecanoylphorbol-13-acetate)-induced acute inflammation, xylene-induced ear edema, cotton pellet–induced granuloma, Freunds’ complete adjuvant (FCA)–induced arthritis, *in vivo* xanthine oxidase assay, LPS-induced peritonitis mouse model, acetic acid–induced vascular permeability assay or measuring writhing reflexes, UV erythema, pleurisy test, etc. (Ferrari et al., 2016; Asante-Kwatia et al., 2020).

### Preclinical Models Used to Investigate Plants Included in this Review

#### *In Vivo* Studies

In this report, carrageenan-induced paw edema is found to be an extensively used *in vivo* model used in 28 investigations. In other studies, paw edema is induced using formalin, egg albumin, and also by exogenous administration of histamine and serotonin. Ear edema models are induced using xylene and croton oil. Cotton pellet–implanted granuloma has been exercised in 12 studies. Other *in vivo* studies include acetic acid–induced writhing test.

#### Paw Edema Model

Carrageenan-induced paw edema is found to be the most extensively used *in vivo* test procedure to screen medicinal plants for their anti-inflammatory activities by Bangladeshi researchers (Supplementary Table S1). This seaweed-derived sulfated polysaccharide is injected to experimental animals such as mouse or rat to induce acute and local inflammation. Paw edema induced by carrageenan is considered to be a highly sensitive, reproducible, and well-established model to investigate anti-inflammatory drugs. This model is characterized by biphasic response. Paw inflammation develops resulting in the synthesis of histamine, serotonin, and bradykinin in the first phase (0–1 h) followed by production of COX-2–mediated prostaglandins and cytokines such as IL-1β, IL-6, IL-10, and TNF-α in the second phase (2–3 h). These mediators can be generated *in situ* either by cellular infiltration or at the site of the local inflammatory insult. The second phase is sensitive to both steroidal and nonsteroidal anti-inflammatory drugs (Posadas et al., 2004; Dzoyem et al., 2017).

Histamine, serotonin, formalin, and egg albumin are injected to obtain paw inflammation in other sets of studies included in this review.

Histamine is a potent inflammatory mediator and is injected to rat or mice paw to induce acute inflammation. This vasoactive amine elicits the release of neuropeptides and prostaglandins from endothelial cells, contributes to neutrophil recruitments, and causes vasodilatation and increased vascular permeability leading to pain and inflammation (Yong et al., 2013b).

Serotonin is another vasoactive amine and inflammatory mediator which increases vascular permeability and can produce acute paw edema *via* direct injection (Yong et al., 2013a).

Formalin-induced paw edema is also a common method to screen medicinal plants for anti-inflammatory activity and is described as a biphasic response in studies. Neurogenic pain develops in the initial phase, and the second phase involves development of inflammatory responses generated by the release of mediators such as histamine, serotonin, bradykinin, prostaglandin, cytokines (IL-1β, IL-6, TNF-α, etc.), and nitric oxide (NO). Formalin-induced paw edema can be modeled for both acute and chronic type of inflammatory assays (John and Shobana, 2012; Arzi et al., 2015).

Paw edema induced by egg albumin is an acute and triphasic phenomenon as described by Dzoyem et al. (2017), where the first phase is mediated by histamine and serotonin and then the bradykinin-mediated second phase is followed by a third phase mediated by cyclooxygenase to produce bradykinin proteases and prostanoids (Dzoyem et al., 2017).

Usually the changes in thickness, volume, weight, and histology of edematous paw induced by any of the abovementioned methods are examined, and by comparison among the control and treated groups, the anti-inflammatory activities of the plant samples are determined (Kim et al., 2006).

#### Ear Edema Model

Xylene-induced ear edema is another *in vivo* model exercised to determine anti-inflammatory activity of plants. Topical administration of xylene causes cutaneous ear inflammation which is acute in nature and is characterized by vasodilatation, cellular infiltration, and edema formation (Lu et al., 2006). Ear edema can be induced by applying croton oil to the ear of rats or mice. This acute inflammatory action is also marked by synthesis of prostaglandins, migration of neutrophils, increased vascular permeability, and development of edema (Luo et al., 2014). In such models, the rats or mice are killed, and circular sections of ears are collected to examine the changes in weight, thickness, and histology profile, which are then compared among the control and treated groups to evaluate the anti-inflammatory activities of the plant samples.

#### Cotton Pellet–Induced Granuloma Model

Cotton pellet–inserted granuloma in rodents is described as a chronic model which is the proliferative phase of inflammation. This pharmacological procedure consists of three phases, namely, 1) transudative, 2) exudative, and, 3) proliferative (Swingle and Shideman, 1972). The granuloma is actually a highly vascularized tissue which is formed at the site of the subcutaneously implanted cotton pellet due to migration of monocytes, proliferation of fibroblasts, increased vascular permeability, and accumulation of fluid and proteinaceous components (Dzoyem et al., 2017). Anti-inflammatory substances can inhibit the growth of granuloma tissue *via* interfering with the phases of granuloma formation.

#### Acetic Acid–Induced Writhing Test

Acetic acid induced writhing is explained as a model of visceral inflammatory pain, generated by releasing endogenous mediators which can trigger nociceptors and are sensitive to nonsteroidal anti-inflammatory drugs and drugs that act on the central nervous system (Bighetti et al., 1999; Jones et al., 2005). Acetic acid is injected into the peritoneal cavity of the experimental animal which causes release of proinflammatory cytokines such as TNF-α, IL-1β, and IL-8 by peritoneal macrophages and mast cells (Ribeiro et al., 2000). As a result, acute inflammatory response arises in the peritoneal area of the experimental animals, and then the animals react with characteristic writhing (Dzoyem et al., 2017). Substances with anti-inflammatory property can inhibit the number of writhes over a time course.

#### *In Vitro* Studies

*In vitro* assays minimize the ethical issues associated with the use of animals in the early phases of drug discovery (Williams et al., 2008). They are mostly cell-based and protein-based and are usually rapid, easy to conduct, and cost-effective, and most importantly, they help understand the molecular mechanism of bioactive compounds to render anti-inflammatory activity (Dzoyem et al., 2017).

RBC membrane stabilization assay is the most extensively used screening tool which has been found to be exercised in 36 studies in this report. Other *in vitro* experiments include protein denaturation, protease inhibition, and direct estimation of lipoxygenase (LOX) inhibition by the plant samples using the LOX inhibition assay.

#### RBC Membrane Stabilization Method

In RBC membrane stabilization assay, the red blood cells (erythrocytes) are exposed to various injurious stimuli which can be thermal (heat), mechanical (hypotonic solution), or chemical (methyl salicylate, phenyl hydrazine, etc.) to induce hemolysis. The RBC membrane has a structure similar to that of the lysosome which is a membrane-bound cellular organelle and can release enzymes (PLA_2_, protease, etc.) capable of inducing inflammatory process. Stabilization of the lysosomal membrane prevents the release of those inflammatory mediators. Therefore, plant extracts or compounds effective in protecting the rupture of the erythrocyte membrane are expected to inhibit the release of inflammatory mediators from the lysosome by stabilizing the lysosomal membrane and hence considered to possess anti-inflammatory activity (Umapathy et al., 2010; Anosike et al., 2012).

#### Protein Anti-Denaturation Assay

Denaturation of protein has also served as an *in vitro* pharmacological method to screen anti-inflammatory activity of plant extracts. Bovine serum albumin (BSA) and egg albumin are commonly used proteins for this purpose. The protein loses its secondary and tertiary structures when exposed to heat or substances such as strong acid or base, concentrated salt solution, or organic solvent. Biologically active proteins usually lose their biological activity upon denaturation (Nirmal and Panichayupakaranant, 2015). As explained by Williams et al*.* (2008), the extracts or molecules which exhibit anti-denaturation property at a very low concentration (ng/ml) should be selected for further drug development processes.

#### Protease Inhibition Assay

Proteases (also called proteinase) are enzymes that catalyze proteolysis, that is, the hydrolysis of peptide bonds, leading to breakdown of proteins (López-Otín and Overall, 2002). Activation of these enzymes can cause tissue damage associated with inflammatory disorders and can be prevented with drugs having protease inhibitory activity (Kumar et al., 2013). Examples of proteases involved in inflammatory reactions include human neutrophil elastase, MMPs (MMP-2 and -9), trypsin etc. (Benedek et al., 2007; Aziz et al., 2015) and are utilized by the researchers to study the *in vitro* anti-inflammatory potential of plants.

### Toxicological Aspect

Toxicology is defined as a branch of science that deals with poisons (Hodgson, 2004), and as stated by Macht (1938), “every drug that is worth anything as a medicinal agent is also a poison.” Toxicity arises due to the interaction between cellular macromolecules and poisons or toxicants (Jothy et al., 2011). Evaluation of toxicity of such agents is, therefore, also crucial besides evaluating their pharmacological properties. To assure the safe medicinal use of plants or plant products, estimation of toxicity is a must. Numerous laboratory procedures have been developed for this purpose.

It can be noticed in the present study that toxicity studies have not been conducted with almost half of the plants (Supplementary Table S1) at the same time when the anti-inflammatory activities are being evaluated. Out of 48 plants, 25 have undergone preliminary assessment of toxicity by at least one of the cited works. The acute toxicity text and brine shrimp (*Artemia salina*) lethality test were found to be the primarily used assay methods to assess the toxicity of plant extracts.

The acute toxicity test is an initial screening step to assess and evaluate toxic properties of substances. LD_50_ which is the dose of the test sample that leads to 50% lethality in the tested group of animals can be determined using this assay (Akhila et al., 2007). Multiple graded doses or a considerably higher single dose than that of the plant extract under investigation were commonly administered to rats or mice to make an estimation of the margin of safety using the acute toxicity test (Supplementary Table S1). Another assay, brine shrimp lethality test, is appraised to be the most convenient method to monitor toxicity of the medicinal plants. This is a rapid and simple predictive tool for toxic potential of plant extracts in humans (Hamidi et al., 2014). Brine shrimp is commonly used as the test organism. LC_50_ which is the concentration of the test sample that leads to 50% lethality in the nauplii is determined usually by using the graph of mean percentage mortality vs. the log of concentration (Syahmi et al., 2010). The *Allium cepa* toxicity test is also a common test used by researchers to investigate the toxicity of various substances. This is a sensitive *in vivo* test method and is used to determine cytotoxic and genotoxic effect of different substances. This test can serve as an indicator of toxicity of the tested samples since it shows good correlation with tests in other living systems. An inhibition of root growth or mitotic index values in the treated onion roots indicates cytotoxic effects, and the chromosomal aberration in the treated onion root tip meristems indicates genotoxic effects of the materials or plant extracts under investigation (Adegbite and Sanyaolu, 2009).

### Overview on Selected Anti-Inflammatory Plants

**FIGURE 2:**
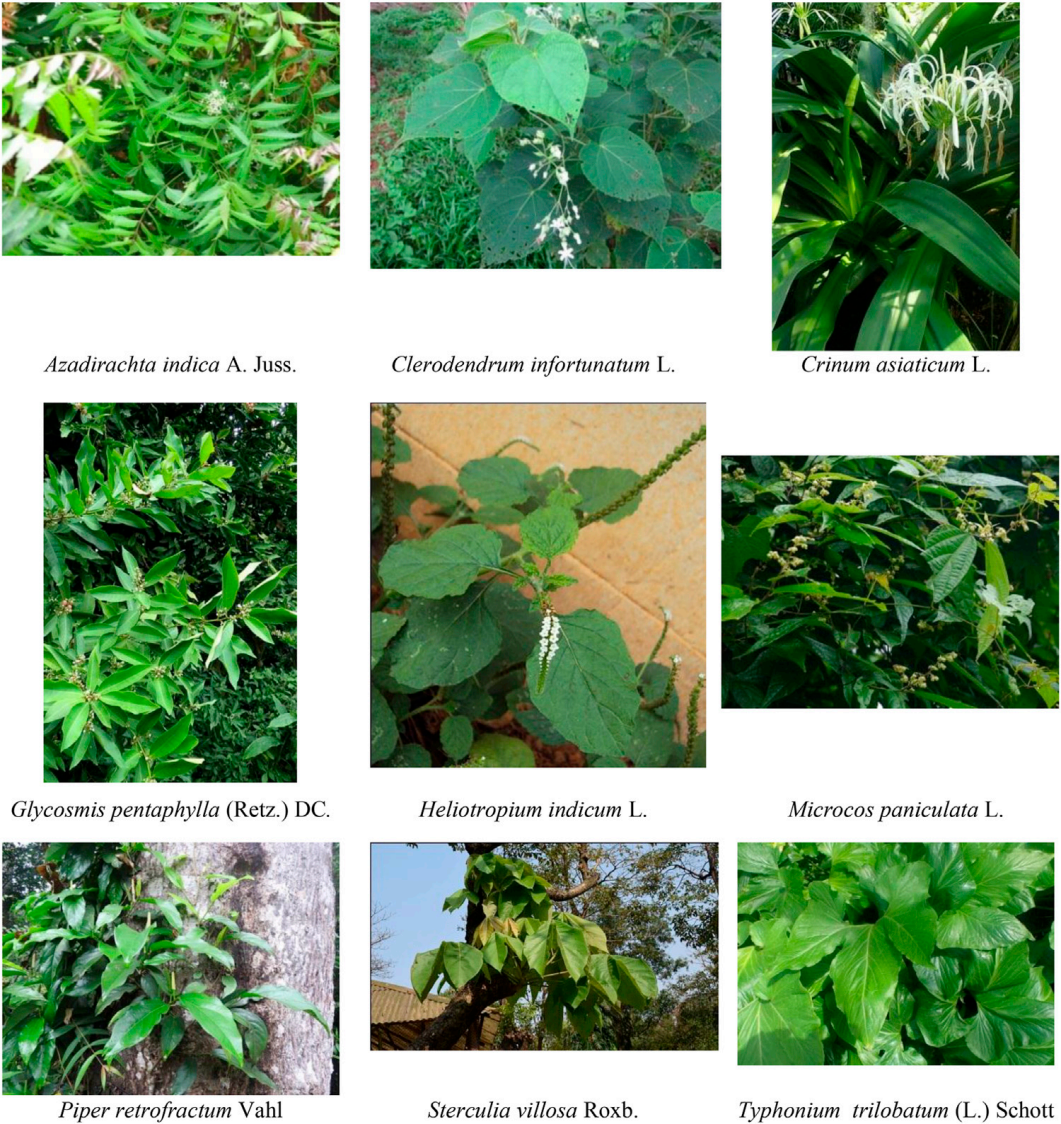
Plants with anti-inflammatory activity, namely, *A. indica*, *C. infortunatum*, *C. asiaticum*, *G. pentaphylla*, *H. indicum*, *M. paniculata, P. retrofractum*, *S. villosa*, *and T. trilobatum* were obtained from Wikimedia Commons under GNU free documentation license (http://en.wikipedia.org/wiki/GNU_Free_Documentation_License), whereas *P. reticulatus* was obtained from the web page “Flora of Bangladesh,” from the Survey of Vascular Flora of Chittagong and the Chittagong Hill Tracts Project, Bangladesh National Herbarium (http://bnh-flora.gov.bd), Ministry of Environment & Forest, People’s Republic of Bangladesh.

Each of the plant included in the present work is useful in traditional medicine to manage inflammation or inflammatory diseases since that was one of the inclusion criteria. However, for ease of discussion, the following nine plants have been selected (Figure 2), which have demonstrated remarkable anti-inflammatory activity in three or more individual reports (Supplementary Table S1). The habitat, similar studies conducted with the species other than the Bangladeshi one, and anti-inflammatory molecules reported from these plants have been overviewed.

#### *Azadirachta indica* A. Juss.

**FIGURE 3:**
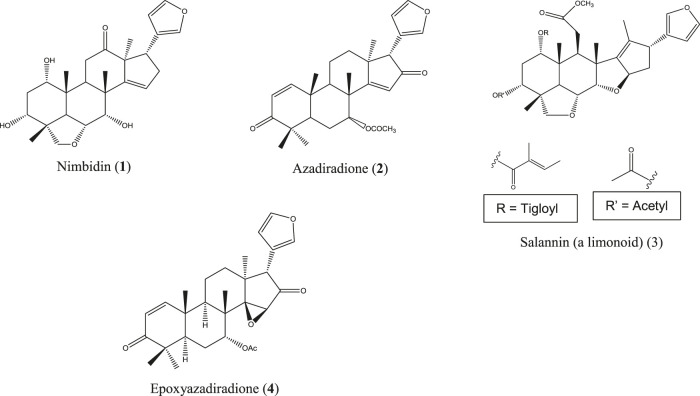
Anti-inflammatory compounds isolated from *A. indica*.

*A. indica* is locally known as neem and is commonly found all over Bangladesh and in India, Pakistan, and Nepal. This herb is used in traditional healing practices, including Ayurvedic, Unani, and Homeopathic systems, to a great extent (Alzohairy, 2016). Though originated from Asia, neem is now cultivated worldwide. Medicinal values, most importantly the anti-inflammatory action of neem extracts and neem compounds, have been reported in a number of studies. Indian neem leaf extract and neem seed oil have inhibited cotton pellet–induced granuloma and carrageenan-induced paw edema, respectively, in rats (Chattopadhyay, 1998; Naik et al., 2014). The Nigerian variety of neem showed pronounced anti-inflammatory activity by reducing carrageenan-induced rat paw edema (Okpanyi and Ezeukwu, 1981). Polysaccharide fractions from the neem seed tegument of Brazil exhibited potent anti-inflammatory activity in acute inflammatory test models (Pereira et al., 2012). However, in another Brazilian study, the ethanolic neem fruit extract did not reduce abdominal edema in the carrageenan-induced inflammatory model of zebrafish and was found to be nontoxic in zebra fish as well as the *A. salina* lethality test (Batista et al., 2018). The neem fruit extract azadirachtin A, purchased in China, markedly reduced the levels of TNF-α, IL-6, IL-1b, TLR4, and NF-κB followed by inhibition of tissue inflammation (He et al., 2020). In a study, conducted in Luxembourg, a strong effect of the neem extract has been observed on proinflammatory cell signaling *via* modulation of the NF-κB pathway (Schumacher et al., 2011). The neem compounds which have significantly attenuated inflammation and related diseases are nimbidin (**1**) (Pillai and Santhakumari, 1981; Kaur et al., 2004), azadiradione (**2**) (Ilango et al., 2013), salannin (**3**), epoxyazadiradione (**4**) (Alam et al., 2012) (Figure 3), and a series of other limonoids (Akihisa et al., 2011; Islas et al., 2020).

#### *Clerodendrum infortunatum* L.

**FIGURE 4:**
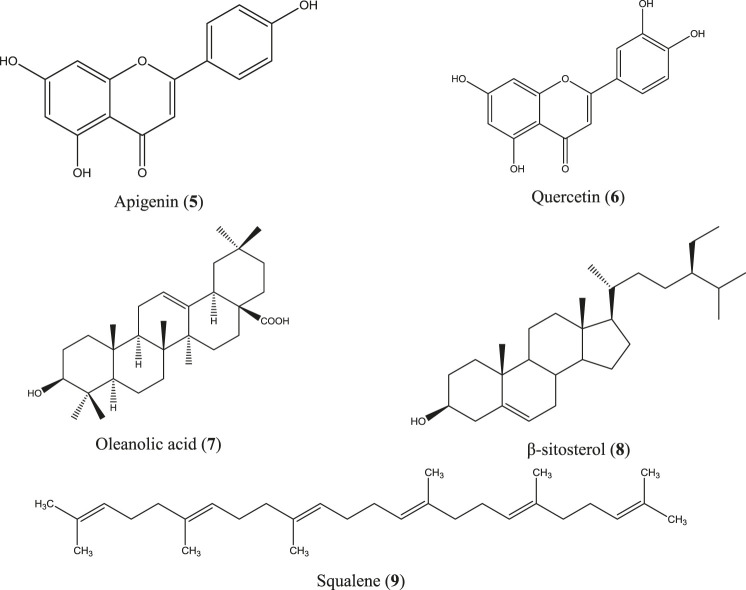
Anti-inflammatory compounds isolated from *C. infortunatum*.

*C. infortunatum* is a shrub and is widely distributed throughout Bangladesh, India, Sri Lanka, Thailand, and Malaysia, and is used in the indigenous systems of medicine, including Ayurveda, Unani, and Homeopathy (Ahmed et al., 2007; Kekuda et al., 2019). Ethanol extract of leaf and aqueous acetone extract of the root bark prepared from the Indian species of this plant have shown anti-inflammatory activity by inhibiting paw edema in rats (Chandrashekar and Rao, 2013; Helen et al., 2018), whereas hydroethanolic extract of the leaf, stem, and root of *C. infortunatum* dose-dependently inhibited NO production in LPS-stimulated macrophage and showed no sign of mortality in the acute toxicity study (Dutta et al., 2018). In another study, both leaf and root extracts were found safe in the *in vivo* experimental model (Nandi et al., 2017). Several compounds have been identified from *C. infortunatum* with important medicinal values (Nandi and Mawkhlieng Lyndem, 2016), the major being 3-deoxy-d-mannoic lactone, glycerin, and xylitol as analyzed using the gas chromatography coupled with mass spectroscopy (GC-MS) technique (Ghosh et al., 2015) along with viscosene and several flavonoid glycosides (Uddin et al., 2020). Anti-inflammatory moieties reported from this plant include apigenin (**5**) (Sinha et al., 1981), quercetin (**6**) (Gupta and Gupta, 2012), oleanolic acid (**7**) (Sannigrahi et al., 2012), β-sitosterol (**8**) (Gupta and Singh, 2012; Paniagua-Pérez et al., 2017), and squalene (**9**) (Figure 4) (Choudhury et al., 2009).

#### *Crinum asiaticum* L.

**FIGURE 5:**
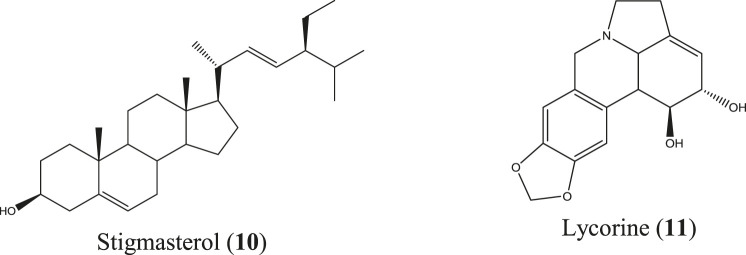
Anti-inflammatory compounds isolated from *C. asiaticum*.

*C. asiaticum* is an evergreen herb found in Bangladesh, India, Sri Lanka, Myanmar, Thailand, Malaysia, China, and Japan. This herb is randomly inhabited in the hilly areas of Bangladesh, especially the Chittagong Hill Tracts, and has widely been used in traditional and Ayurvedic systems of medicines (Rahman et al., 2013; Sharma et al., 2020). The Malaysian species of this plant caused significant reduction of mice paw edema (Samud et al., 1999) and prevented new blood vessel formation from the aortic ring explants, exhibiting potential anticancer activity known to be influenced by inflammation (Yusoff et al., 2017). The list of compounds isolated from this plant is also long and includes lycorine, crinamin, stigmasterol, cycloartenol, etc. (World Health Organization, 1998), among which stigmasterol (**10**) (Al-Attas et al., 2015) and lycorine (**11**) (Figure 5) (Çitoğlu et al., 1998; Saltan Çitoğlu et al., 2012) are reported to be anti-inflammatory molecules. Crinamin, a protein isolated from the latex of *C. asiaticum* leaf exhibited anti-inflammatory activity by reducing carrageenan-induced paw edema and cotton pellet–induced granuloma in rats and also showed protein anti-denaturation and RBC membrane stabilization activity (Rai et al., 2018).

#### *Glycosmis pentaphylla* (Retz.) DC.

**FIGURE 6:**
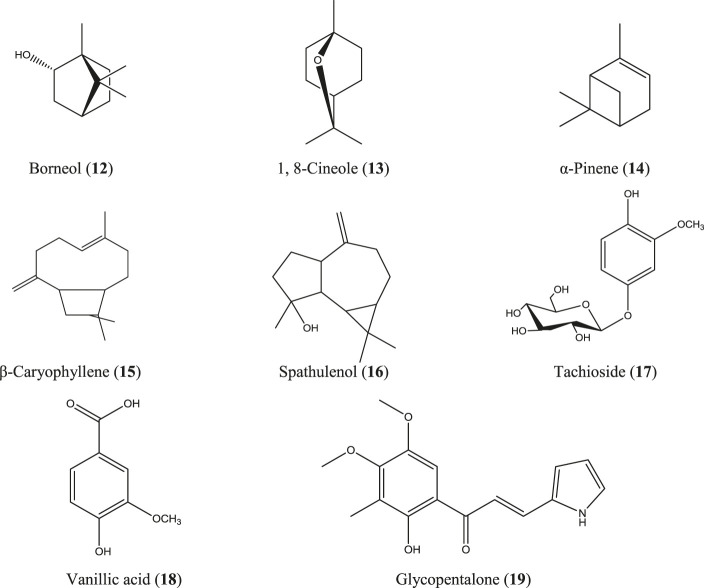
Anti-inflammatory compounds isolated from *G. pentaphylla*.

*G. pentaphylla* is a shrub or small tree that grows at low altitudes of Bangladesh, India, Malaysia, southern China, and the Philippine Islands (Hossain S. M. et al., 2012). Root extract from the same Indian plant inhibited carrageenan- and egg albumin–induced paw edema, xylene-induced ear edema, and formalin-induced arthritic inflammation in rats (Rao and Raju, 2009; Arora and Arora, 2016). The essential oils obtained from the bark, leaf, and seed of *G. pentaphylla* are enriched with numerous bioactive constituents (Ahmed et al., 2000), and some of them have well-documented anti-inflammatory activity such as borneol (**12**) (Zou et al., 2017), 1, 8-cineole (**13**) (Santos and Rao, 2000), α-pinene (**14**) (Kim DS. et al., 2015; Özbek and Yılmaz, 2017), caryophyllene (**15**), caryophyllene oxide (Tung et al., 2008), and spathulenol (**16**) (Figure 6) (do Nascimento et al., 2018). In studies in China, a number of prenylated sulfur-containing amides and a phenolic glycoside (tachioside) (**17**) (Figure 6) isolated from this plant also exhibited anti-inflammatory activity *via* downregulation of nitric oxide (NO) production in LPS-stimulated RAW 264.7 macrophages (Tian et al., 2014; Nian et al., 2020). Numerous other compounds have been isolated from this plant, including anti-inflammatory stigmasterol (**10**) (Figure 5), vanillic acid (**18**) (Siddiqui et al., 2019; Soma et al., 2019; Chokchaisiri et al., 2020; Ziadlou et al., 2020), and glycopentalone (**19**) (Figure 6) (Sreejith and Asha, 2015; Sasidharan and Vasumathi, 2017).

#### *Heliotropium indicum* L.

*H. indicum* is a perennial herb indigenous to Bangladesh, India, Sri Lanka, Nepal, and Thailand and also grows in some parts of Africa (Samira et al., 2016). The Indian *H. indicum* has been found to attenuate carrageenan-, egg white–, and formalin-induced paw edema and cotton pellet–inserted granuloma in rats (Srinivas et al., 2000; Betanabhatla et al., 2007; Ramamurthy et al., 2009; Shalini and Shaik, 2010), whereas the Thai and Ghanaian species have been reported to inhibit proinflammatory mediators in LPS-stimulated RAW 264.7 macrophages (Chunthorng-Orn et al., 2016; Kyei et al., 2016). Analyses on volatile oil composition of this plant have been carried out where phenylacetaldehyde and phytol were found to be the major constituents, and a number of pyrrolizidine alkaloids have also been reported from *H. indicum* (Birecka et al., 1984; Machan et al., 2006; Ogunbinu et al., 2009). In another GC-MS analysis of essential oil, it was revealed that this plant is rich in phenyl derivatives, where methyl salicylate is the major constituent (Joshi, 2020), and interestingly, a small concentration of methyl salicylate is used in topical preparations for the treatment of mild aches and pains of arthritis (Martin et al., 2004).

#### *Microcos paniculata* L.

**FIGURE 7:**
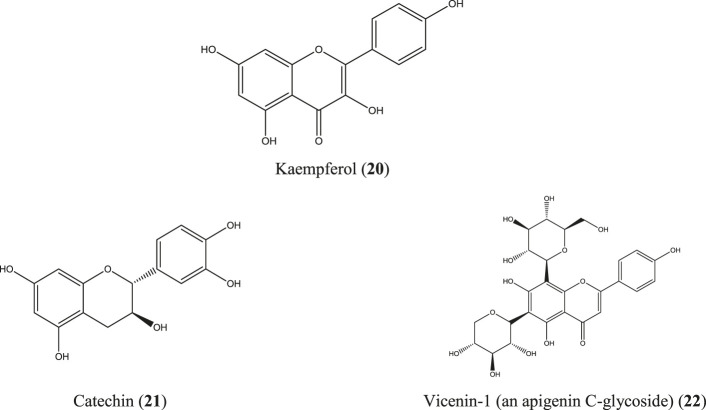
Anti-inflammatory compounds isolated from *M. paniculata*.

*M. paniculata* is a shrub native to southern China, Southeast Asia, and South Asia*.* In Bangladesh, this plant is common at the Sylhet and Chittagong divisions (Al-Amin Sarker et al., 2016; Jiang and Liu, 2019). Besides numerous traditional uses, a diverse range of compounds have been identified from different parts of the plant which include some well-documented anti-inflammatory molecules such as apigenin (**5**), quercetin (**6**) (Figure 4), kaempferol (**20**), catechin (**21**), epicatechin (Perez, 2001; García-Mediavilla et al., 2007; Hämäläinen et al., 2007; Kim SH. et al., 2015; Lesjak et al., 2018), and apigenin C-glycosides (e.g., vicenin-1) (**22**) (Li et al., 2018) (Figure 7). A total flavonoid glycoside fraction obtained from Chinese *M. paniculata* demonstrated potential anti-inflammatory activity by reducing xylene-induced mice ear edema and suppressing proinflammatory cytokines (IL-6, IL-1β, and TNF-α) in LPS-stimulated RAW 264.7 macrophages. In the same study, ten flavone glycosides have been characterized from the total flavonoid glycoside fraction (Yuan et al., 2019).

#### *Piper retrofractum* Vahl

*P. retrofractum* is a flowering vine native to South and Southeast Asia including Bangladesh, India, Malaysia, Indonesia, Singapore, and Sri Lanka. This plant is used as a culinary herb in some regions of Bangladesh and is commonly known as Choi or Chui jhal (Islam MT. et al., 2020). Methanolic leaf extract of the same Indian species has been investigated using chronic anti-inflammatory study models, including carrageenan- and dextran-induced paw edema and an acute model of cotton pellet–induced granuloma (Vaghasiya et al., 2007). *P. retrofractum* fruit extract is an ingredient of a Thai traditional medicine “Benjakul” which has demonstrated strong anti-inflammatory activity in *in vitro* and *in vivo* test systems (Kuropakornpong et al., 2020). In another Thai study, the ethanolic fruit extract of *P. retrofractum* inhibited ethyl phenylpropiolate–induced ear edema and carrageenan-induced paw edema in rats (Sireeratawong et al., 2012). Chabamides, piperine, piplartine, etc. have been reported from this plant (Islam MT. et al., 2020). Essential oil composition of the fruit oils of *P. retrofractum* has also been analyzed, which contains 5.2% of β-caryophyllene (**15**) (Figure 6) (Tewtrakul et al., 2000).

#### *Sterculia villosa* Roxb.

**FIGURE 8:**
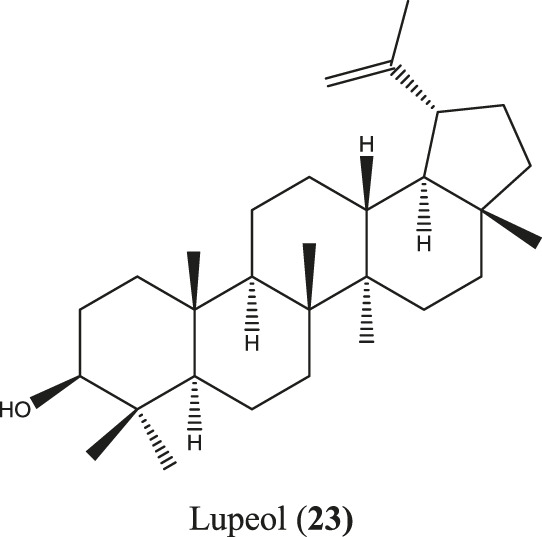
Anti-inflammatory compounds isolated from *S. villosa*.

*S. villosa* is a deciduous small to large tree found in tropical and subtropical countries, including Bangladesh, India, Sri Lanka, and southern China (Hossain M. K. et al., 2012; Hossain et al., 2016). This plant has received special importance for its anti-inflammatory ethnomedicinal value besides many other traditional uses (Namsa et al., 2009; Thabet et al., 2018). Along with different bioactive molecules such as chrysoeriol, chrysoeriol 7-O-β-D-glucoside, diosmetin, etc., anti-inflammatory lupeol (**23**) (Figure 8) has also been reported from *S. villosa* (Seetharaman, 1990; Das et al., 2017).

#### *Typhonium trilobatum* (L.) Schott

*T. trilobatum* or Bengal arum is a perennial herb native to Asia, including Bangladesh, India, and to South Pacific. This is a soft plant that grows in the damp and moist places in Chittagong, Sylhet, and other areas of Bangladesh (Ghani, 2003; Haldar et al., 2011). This plant has plenty of traditional uses and has been evaluated in several studies for its anti-inflammatory action (Supplementary Table S1), but individual phytoconstituents from this plant have not been documented properly yet (Manna et al., 2016).

In addition to the abovementioned nine species, the following species have been found to be studied tremendously in recent years for anti-inflammatory activities and been reviewed here in brief.

#### *Ageratum conyzoides* (L.) L.

*A. conyzoides* is an annual herb with a long history of uses in traditional medicine in the tropical and subtropical countries (Kotta et al., 2020). This plant is very common in West Africa, South America, and some parts of Asia, including Bangladesh (Okunade, 2002; Hassan et al., 2012). Crude extract, organic fractions, and isolated compounds (5′- ethoxynobiletin, 1,2-benzopyrone and eupalestin) from the Brazilian variety of this plant inhibited carrageenan-induced pleurisy in mice by reducing the concentration of myeloperoxidase (MPO), adenosine deaminase (ADA), and NO, and by inhibiting several proinflammatory mediators (e.g., IL-17A, IL-6, TNF-α, and IFN-γ). The isolated compounds also reduced phosphorylation of NF-κB and MAPK (Vigil de Mello et al., 2016). In another study conducted in Brazil, the ethanol extract of the standardized polymethoxyflavone extract of this plant exhibited anti-inflammatory activity in mice (Faqueti et al., 2016), whereas Indonesian *A. conyzoides* leaf extract decreased TNF-α and MMP-9 levels in monosodium iodoacetate–induced osteoarthritis in rats, and anti-inflammatory quercetin (**6**) was detected in the extract (Bahtiar et al., 2017).

#### *Eclipta prostrata* (L.) L.

*E. prostrata* is an annual herb and a common weed native of Asia but is now widely distributed in tropical and sub-tropical areas over the world (Chung et al., 2017; Feng et al., 2019; Yu et al., 2020). In Asia, this plant grows in Bangladesh, India, Indonesia, Cambodia, Malaysia, Nepal, Pakistan, the Philippines, Sri Lanka, Thailand, Vietnam, China, Japan, and Korea (Uddin et al., 2015). The Korean variety of *E. prostrata* ethanolic extract prepared conventionally and using ultrasound as well as wedenolactone (a compound isolated from this plant) exhibited anti-inflammatory activity by reducing IL-6, TNF-α, and PGE_2_ levels (Lee, 2017). In another investigation, a series of flavonoid, flavonoid derivative, triterpenoid, and triterpenoid glycosides were isolated from this plant which reduced the NO level in LPS-stimulated RAW 264.7 cells in varying degrees, where 7-*O*-methylorobol-4′-*O*-*β*-D-glucopyranoside was found to be the most potent compound and reported as an inhibitor of IκB phosphorylation (Le et al., 2020). The aqueous seed extract of the Sri Lankan variety of this plant dose-dependently inhibited denaturation of egg albumin (Kannadas et al., 2020).

#### *Lawsonia inermis* L.

*L. inermis* commonly known as henna has plenty of traditional uses (Supplementary Table S1). This evergreen shrub is mainly cultivated for cosmetic purposes and also for its use in traditional medicine all over the world, but the plant is native to tropical and subtropical countries such as Bangladesh, India, Sri Lanka, and the Middle East (Ghani, 2003; Sharma et al., 2016). Flavonoids isolated from the Indian species of this plant inhibited carrageenan-induced rat paw edema (Manivannan et al., 2015), whereas the flower extract from the Tunisian species inhibited 5-LOX (Chaibi et al., 2015). The ethanol extract from the leaves of this plant reduced formalin-induced (Humaish, 2017) and carrageenan-induced (Vijayaraj and Kumaran, 2018) rat paw edema in studies conducted in Iraq and India. respectively. The Nigerian *L. inermis* leaf extract prepared with n-butanol and ethylacetate reduced carrageenan-induced foot edema in chicks (Chuku et al., 2020). Additionally, a topical formulation prepared from Iranian henna leaves improved contact dermatitis in patients using lower limb prosthetics (Niazi et al., 2020).

#### *Manilkara zapota* (L.) P.Royen

*M. zapota* is an evergreen tree thought to originate from Mexico, Central America, and West Indies and is cultivated throughout Bangladesh and India (Ganguly et al., 2013; Yong and Abdul Shukkoor, 2020). The leaf extract of Indian *M. zapota* inhibited hemolysis of the RBC membrane (Sravani et al., 2015) and inhibited PLA_2_ and 5-LOX, and carrageenan-induced rat paw edema in *in vitro* and *in vivo* experimental models, respectively (Konuku et al., 2017). Apigenin-7-O-β-D-glucuronide methyl ester isolated from Ethiopian *M. zapota* leaves downregulated COX-2 mRNA expression in MCF-7 breast cancer cells (Kamalakararao et al., 2018b) and also inhibited NO and PGE_2_ production in LPS-induced RAW 264.7 macrophages (Kamalakararao et al., 2018a). The fruit extract prepared from the Thai variety exhibited anti-inflammatory potential by inhibiting the TNF-α level in LPS-activated human PMBC cells (Leelarungrayub et al., 2019). Again, several prenylated coumarins identified from Chinese *M. zapota* fruit suppressed NO production in LPS-activated RAW 264.7 cells (Liu et al., 2019).

#### *Withania somnifera* (L.) Dunal

*W. somnifera* is an evergreen woody shrub commonly known as winter cherry or Indian ginseng distributed in tropical and subtropical regions of the world which include Bangladesh, India, Pakistan, Afghanistan, Sri Lanka, China, Egypt, Morocco, Jordan, Congo, and South Africa. This plant is extensively used in Unani and Ayurvedic systems of medicine (Shahriar et al., 2014; Dar et al., 2015; Dar and Ahmad, 2020). Several studies conducted with the Indian species of this plant exhibited significant anti-inflammatory activities. For example, root extract of *W. somnifera* exhibited acute and chronic anti-inflammatory activity in carrageenan-induced rat paw edema and Freund’s adjuvant–induced arthritis models, respectively (Giri, 2016), as well as in collagen-induced arthritic rats (Khan et al., 2019). Leaf extract demonstrated anti-neuroinflammatory activity by attenuating TNF-α, IL-1β, IL-6, RNS, and ROS production *via* the inhibition of NF-κB, AP-1, JNK, and MAPK pathways (Gupta and Kaur, 2016; 2018). Fatty acids from seeds inhibited the release of proinflammatory cytokines (IL-6 and TNF-α) and reduced NF-κB expression and were found to reduce edema besides repairing psoriatic lesions in the TPA-induced psoriatic mouse model. In the same study, the fatty acid components were identified as linoleic acid, oleic acid, palmitic acid, stearic acid, 11,14,17-eicosatrienoic acid, and nervonic acid along with other unidentified components (Balkrishna et al., 2020).

The brief analyses of the abovementioned plants again signify the values of Bangladeshi medicinal plants to be explored for novel anti-inflammatory molecules. However, the reported anti-inflammatory activities of the plants investigated in Bangladesh are primarily based on the anti-inflammatory activity of their crude organic extracts or partitionates. Therefore, a great detail is yet to be unveiled of these plants regarding the purification and characterization of their chemical constituents, which requires different separation techniques and spectroscopic analyses to pinpoint the anti-inflammatory molecules.

According to literature, a huge number of Bangladeshi medicinal plants are used in traditional medicines to combat inflammation and inflammatory disorders. However, due to the set criteria of plant selection, a limited number of plants have been summarized in the present study. The plants reviewed here are useful in traditional medicine to manage inflammation or inflammatory diseases such as arthritis, asthma, hepatitis, psoriasis, bronchitis, and tumor. The anti-inflammatory activities of these plants have been evaluated scientifically using different assay models. The plant samples were administered usually in graded doses of the extract or as a single dose of each of different organic fractions. Thus, in case of graded doses, plant extracts were found to inhibit inflammation in a dose-dependent manner, and in case of different organic fractions, a single dose of different samples demonstrated varying degrees of activity. The significance of activity was assessed in comparison with positive controls. Acetyl salicylic acid, diclofenac sodium, indomethacin, ibuprofen, phenylbutazone, hydrocortisone, etc. were commonly used as positive control drugs for these experiments.

Selected plants, namely, *A. indica*, *C. infortunatum*, *C. asiaticum*, *G. pentaphylla*, *H. indicum*, *M. paniculata*, *P. retrofractum*, *S. villosa*, and *T. trilobatum* were focused in further detail. In addition, *A. conyzoides*, *E. prostrata*, *L. inermis*, *M. zapota*, and *W. somnifera* have been reviewed briefly. Most of these plants have been found to be studied for the same purpose in other Asian countries, including India, Sri Lanka, Thailand, Malaysia, China, Korea, and Indonesia along with countries such as Brazil, Nigeria, Ghana, Ethiopia, Iraq, Iran, and Luxembourg where these plants have been proved again to have potential for anti-inflammatory activities. A number of well-documented anti-inflammatory molecules, for example, apigenin (**5**), quercetin (**6**), 1,8-cineole (**13**), *α*-pinene (**14**), *β*-caryophyllene (**15**), kaempferol (**20**), catechin (**21**), and apigenin C-glycosides (e.g., vicenin-1) (**22**) have been characterized from these plants.

Although attributed for diverse beneficial effects with a rich source of therapeutic agents’ plants have toxicological properties as well. The acute toxicity test has been conducted by many of the researchers to determine dose ranges for subsequent pharmacological assays and to observe changes in behavior that is signs such as restlessness, respiratory distress, general irritation, coma, convulsion, locomotor ataxia, diarrhea, and weight loss elicited by the plant samples under investigation. The brine shrimp lethality test is also used by the investigators to estimate toxicity of plant extracts. LC_50_ values of the extracts have been determined using this assay. Higher LC_50_ values indicate higher margin of safety, whereas samples with lower LC_50_ values are considered to be toxic and often recommended to be evaluated for anticancer properties. However, crude extract from plants usually is a combination of few or many biologically active constituents. Thus, the compound(s) demonstrating useful pharmacological activity may or may not be responsible for the elicited toxicity. Therefore, identifying the active constituent and evaluation of toxicity of that active constituent is highly crucial for these traditionally important anti-inflammatory plants of Bangladesh.

## Concluding Remarks

This review indicates that the medicinal plants of Bangladesh have immense significance for anti-inflammatory activity and have the potential to contribute toward the discovery and development of novel therapeutic approaches to combat inflammatory disorders which are the leading cause of innumerable human diseases all over the globe. Though possessing traditional significance to manage inflammatory diseases, these plants chiefly underwent preliminary screening for anti-inflammatory effects and require intensive research studies, including identification of active constituents, understanding the mechanism of action precisely, evaluation for safety and efficacy, and formulation of safe and effective drug products.

These early-stage screenings of Bangladeshi medicinal plants were mostly carried out for academic purposes, and there was hardly any industrial support behind these projects. Proper industrial collaboration can forge the aforementioned stages of exploration and can thereby contribute to extend the anti-inflammatory therapeutic armory.

## Glossary

ADA: adenosine deaminase
AA: arachidonic acid
AP-1: activator protein-1
BSA: bovine serum albumin
CD: cluster of differentiation
COX: cyclooxygenase
ELISA: enzyme-linked immunosorbent assay
ERK: extracellular signal–regulated kinase
FCA: Freund’s’ complete adjuvant
GC-MS: gas chromatography–mass spectroscopy
GI: gastrointestinal
HETE: hydroxyleicosatetraenoic acid
IBD: inflammatory bowel disease
ICAM-1: intercellular adhesion molecule-1
IκB: inhibitory κB
IL: interleukin
INF-γ: interferon-γ
IP: intraperitoneal
JAK: Janus kinase
JNK: c-Jun-N-terminal kinase
LOX: lipoxygenase
LC_50_: lethal concentration
LD_50_: lethal dose
LTB4: leukotriene B4
LTC4: leukotriene C4
LPS: lipopolysaccharide
MMP: matrix metalloproteinase
MAPKs: mitogen-activated protein kinases
MPO: myeloperoxidase
NF-κB: nuclear factor-κB
ng: nanogram
NO: nitric oxide
NSAIDs: nonsteroidal anti-inflammatory drugs
PAF: platelet-activating factor
PKC: protein kinase C
PLA2: phospholipase A2
PBMCs: peripheral blood mononuclear cells
PGE2: prostaglandin E2
P.O.: per oral
RBC: red blood cell
RNS: reactive nitrogen species
ROS: reactive oxygen species
SAIDs: steroidal anti-inflammatory drugs
SOD: superoxide dismutase
STAT: signal transducer and activator of transcription
TNF-α: tumor necrosis factor-α
TPA: 12-O-tetradecanoylphorbol-13-acetate
TXA2: thromboxane A2
VCAM-1: vascular cell adhesion molecule-1
