# Supplementary material for: Anti-Inflammatory Medicinal Plants of Bangladesh—A Pharmacological Evaluation
Source: Front Pharmacol. 2022 Mar 24;13:809324. doi: 10.3389/fphar.2022.809324 (PMC8987533; doi:10.3389/fphar.2022.809324)
Supplement: Supplementary file 1 [file Table1.docx]

**Table S1** Medicinal plants of Bangladesh with reported anti-inflammatory activities

| **Plant name (Family) - Common or English name (Local name)** | **Traditional use(s)** | **Study type** | **Plant part (s) and concentration/ dosage** | **Control** | **Result** | **Toxicity of the same active extract/ sample** | **Reference(s)** |
| --- | --- | --- | --- | --- | --- | --- | --- |
| *Acanthus ilicifolius* L. (Acanthaceae) - Mangrove Holly (Hargoja) | **Leaf:** as expectorant and as fomentation in rheumatism and neuralgia; with tender shoots used for snakebite. **Root:** in the treatment of paralysis, asthma, cough, leucorrhea and debility; decoction is used for gargling in sore mouth and toothache ([Ghani, 2003](#_ENREF_95)) | *In vitro,* human RBC membrane stabilization, LOX inhibition assay | Leaf  50-800 μg/mL of ethanol extract for RBC membrane stabilization, whereas 6.25-100 μg/mL for LOX inhibition assay | Positive control: 50-800 μg/mL of ascorbic acid and diclofenac sodium for H_2_O_2_ and hypotonicity induced hemolysis and 6.25-100 μg/mL quercetin for LOX inhibition assay | *A. ilicifolius* inhibited H_2_O_2_ and hypotonicity induced hemolysis as well as LOX in a concentration dependent manner | Not determined | ([Biswas et al., 2019](#_ENREF_50)) |
| *Acmella paniculata* (Wall. ex DC.) R.K.Jansen or  *Spilanthes calva* DC. or *S. paniculata* Wall. Ex DC. (Asteraceae) - Toothache plant (Surza konna or Shormoni) | **Flower:** tincture is used to relieve toothache and is efficacious in throat infection and paralysis of the tongue. **Root**: purgative. **Seed:** stimulant and sialagogue  Decoction of this plant is also credited for diuretic and lithotriptic action and is used in the treatment of dysentery, rheumatism, scabies and psoriasis ([Ghani, 2003](#_ENREF_95)) | *In vitro*, mice RBC membrane stabilization assay;  *In vitro*, human RBC membrane stabilization assay | Whole plant  2 mg/mL of each of methanol extract and its petroleum ether, carbon tetrachloride, dichloromethane, ethyl acetate and aqueous fraction;  Leaf  1 mg/mL of each of ethanol extract and its *n*-hexane and ethyl acetate fraction | Positive control: acetyl salicylic acid 0.10 mg/mL;  Positive control: acetyl salicylic acid 0.10 mg/mL | 2 mg/mL of extract and fractions significantly inhibited lysis of mice RBC membrane induced by hypotonic solution;  1 mg/mL of extract and fractions inhibited lysis of human RBC membrane induced by hypotonic solution and heat to various extent | Not determined;  **Brine shrimp lethality test:** LC_50_ values of 48.98 and 216.77 μg/mL were obtained with the *n*-hexane and ethyl acetate fractions respectively | ([Sikder et al., 2010](#_ENREF_262))  ([Hossain et al., 2014b](#_ENREF_129)) |
| *Aegiceras corniculatum* (L.) Blanco (Myrsinaceae) - Black mangrove or river mangrove (Khalisha) | **Bark:** used in the treatment of inflammation, asthma, painful arthritis and rheumatism ([Uddin, 2019](#_ENREF_292)) | *In vitro,* human RBC membrane stabilization, LOX inhibition assay | Leaf and bark For both leaf and bark 50-800 μg/mL of ethanol extract for RBC membrane stabilization, whereas 6.25-100 μg/mL for LOX inhibition assay | Positive control: 50-800 μg/mL of ascorbic acid and diclofenac sodium for H_2_O_2_ and hypotonicity induced hemolysis and 6.25-100 μg/mL quercetin for LOX inhibition assay | Both leaf and bark extract from *A. corniculatum* inhibited H_2_O_2_ and hypotonicity induced hemolysis as well as LOX in a concentration dependent manner | Not determined | ([Biswas et al., 2019](#_ENREF_50)) |
| *Ageratum conyzoides* (L.) L. (Asteraceae) - Appa grass (Fulkuri or Uchanti)  ([Ghani, 2003](#_ENREF_95)) | **Leaf:** used in cuts, sores, boils, skin diseases and have antilithic property; decoction is used in diarrhea, dysentery, colic, rheumatism and externally in ague. **Root:** used in cuts and sores; antilithic ([Ghani, 2003](#_ENREF_95)) | *In vivo*, carrageenan and histamine induced paw edema;  Carrageenan induced paw edema | Leaf  P.O. 200 and 400 mg/kg body wt of ethanol extract;  Whole plant P.O. 0.5, 1.0 and 1.5 g/kg body wt of ethanol extract | Positive control: P.O. indomethacin 10 mg/kg body wt and negative control: P.O. 2% Tween 80 in normal saline 2 mL/kg body wt;  Positive control: P.O. diclofenac sodium 40 mg/kg body wt and negative control: P.O. distilled water | 400 mg/kg of ethanol extract inhibited paw edema significantly in the time of 4 h after injection with carrageenan and histamine;  1.0 and 1.5 g/kg of ethanol extract inhibited paw edema significantly in a time and concentration dependent manner in the time of 4 h after injection with carrageenan | **Acute toxicity test**: The extract was administered in graded doses of 200, 400, 800, 1,600 and 3,200 mg/kg body wt as single oral dose and was found safe up to 3,200 mg/kg since no mortality or toxic reaction was recorded in any group after 48 h of administration;  **Acute toxicity test**: The extract was administered in graded doses of 0.5, 1.0, 2.0, 3.0 and 4 g/kg body wt as single oral dose, observed closely for first 24 h and also next 14 days for delayed toxic effect. No mortality or toxicity was noted | ([Hassan et al., 2012](#_ENREF_117))  ([Rahman et al., 2012](#_ENREF_225)) |
| *Aglaia cucullata* (Roxb.) Pellegr. or  *Amoora cucullata* Roxb. (Meliaceae) - Pacific maple (Amur, latmi or natmi) | **Leaf:** used to treat inflammation; juice is antibacterial and used for the treatment of dysentery, skin diseases and cardiac diseases ([Das et al., 2005](#_ENREF_68)) | *In vivo*, carrageenan induced paw edema and cotton pallet induced granuloma | Leaf  P.O. 200 and 400 mg/kg body wt of methanol extract | Positive control: P.O. aspirin 150 mg/kg body wt and negative control: P.O. 1% Tween 80 in water 10 mL/kg of body wt | 200 and 400 mg/kg body wt of methanol extract inhibited paw edema significantly in a dose dependent manner where maximum inhibition exerted at 3 h after injection with carrageenan and these doses significantly reduced the weight of cotton pallets | Not determined | ([Das et al., 2005](#_ENREF_68)) |
| *Alangium salviifolium* (L.f.) Wangerin (Alangiaceae) - Sage-leafed alangium (Akarkanta or Ankora)  ([Ghani, 2003](#_ENREF_95)) | **Root bark:** anthelmintic, purgative, emetic and febrifuge, powered root bark is used against poisonous bites. **Fruit:** used in consumption and hemorrhages.  Other uses of various parts include leprosy, skin disease, dropsy, rheumatic pain, stomach ache, cholera, phthisis and bronchitis ([Ghani, 2003](#_ENREF_95)) | *In vivo*, carrageenan and formalin induced paw edema | Flower  P.O. 50 and 100 mg/kg body wt of both methanol extract and its chloroform fraction | Positive control: P.O. indomethacin 10 mg/kg body wt and negative control: P.O. 1% Tween 80 | 50 and 100 mg/kg body wt of both methanol extract and its chloroform fraction inhibited paw edema significantly in a dose dependent manner in the time of 4 h after injection with carrageenan and formalin | Not determined | ([Zahan et al., 2013](#_ENREF_315)) |
| *Alocasia macrorrhizos* (L.) G.Don or *Alocasia indica* (Lour.) Spach (Araceae) - Giant alocasia, metallic taro (Mankachu) | **Leaf:** used as digestive, laxative, diuretic, astringent and to treat rheumatic arthritis; styptic and used to treat tumors. **Root:** is useful against inflammations, diseases of the abdomen and spleen and in piles. **Rhizome:** decoction is used for abdominal pain and vomiting; used as rubefacient, external stimulant and for fevers ([Rahman et al., 2011b](#_ENREF_228))  **Tuber:** ash from burning tuber is used to cure lacerations and infections of tongue and mouth ([Ghani, 2003](#_ENREF_95)) | *In vivo*, carrageenan induced paw edema;  *In vitro,* human RBC membrane stabilization assay | Rhizome  P.O. 300 and 600 mg/kg body wt of ethanol extract;  1 mg/mL of each of methanol extract and its petroleum ether, carbon tetrachloride, chloroform and aqueous fraction | Positive control: P.O. aspirin 150 mg/kg body wt and negative control: P.O. 1% Tween 80 in water 10 mL/kg of body wt;  Positive control: acetyl salicylic acid 0.10 mg/mL | 300 and 600 mg/kg body wt of ethanol extract inhibited paw edema significantly in the time of 5 h after injection with carrageenan;  1 mg/mL of methanol extract and fractions significantly protected the lysis of human erythrocyte membrane induced by heat and hypotonic solution | Not determined;  **Brine shrimp lethality test**: LC_50_ values of 5.39, 0.58, 0.50, 8.85 and 77.64 μg/mL were obtained with methanol extract and its petroleum ether, carbon tetrachloride, chloroform and aqueous fractions respectively | ([Rahman et al., 2011b](#_ENREF_228))  ([Hoque et al., 2011](#_ENREF_124)) |
| *Argyreia argentea* (Roxb.) Sweet  (Convolvulaceae) - (Bitarak, rupar tola ludi)  Status: ambiguous | **Leaf:** fomentation with heated leaves is applied on boils; fresh juice is given to cure marasmus and spermatorrhea; pills made from leaves are given in paralysis ([Uddin, 2006](#_ENREF_293))  **Leaf**: paste is used to treat boils; useful to treat gastric problems, paralysis, arthritis, cold and painful sensation ([Uddin, 2019](#_ENREF_292)) | *In vivo*, carrageenan induced paw edema | Leaf  P.O. 0.5, 1.0 and 1.5 g/kg body wt of methanol extract;  Stem  P.O. 1.0, 1.5 and 2.0 g/kg body wt of methanol extract | Positive control: P.O. diclofenac sodium 40 mg/kg body wt and negative control: P.O. distilled water | 0.5, 1.0 and 1.5 mg/kg body wt of methanol extract inhibited paw edema significantly in a dose dependent manner in the time of 4 h after injection with carrageenan;  1.0, 1.5 and 2.0 mg/kg body wt of methanol extract inhibited paw edema significantly in a time dependent manner in the time of 4 h after injection with carrageenan | **Acute toxicity test:** The extract was administered in graded doses of 0.5, 1.0, 2.0, 3.0 and 4 g/kg body wt as single intraperitoneal injection, observed closely for first 24 h and also next 10 days for delayed toxic effect. No mortality or toxicity was noted ;  Not determined | ([Uddin et al., 2010](#_ENREF_289))  ([Dina et al., 2010](#_ENREF_75)) |
| *Azadirachta indica* A.Juss. (Meliaceae) - Indian lilac  (Neem or nim gachh)  ([Ghani, 2003](#_ENREF_95)) | **Leaf:** antiseptic and used in boils, ulcer, eczema, ringworm and scabies. **Flower:** atonic dyspepsia and general debility. **Gum:** demulcent tonic and is efficacious in catarrhal affections. **Seed:** oil from seed in used in ulcer, chronic skin disease and rheumatism. **Leaf, bark, gum and seed:** extracts used in scorpion sting and snake bite and for anti-viral, antineoplastic and antifungal properties ([Ghani, 2003](#_ENREF_95)) | *In vivo*, formalin induced paw edema;  Carrageenan induced paw edema;  Carrageenan induced paw edema and cotton pallet induced granuloma | Leaf  IP 400 mg/kg of body wt of aqueous extract;  Bark  P.O. 250 and 500 mg/kg of body wt of ethanol extract;  Leaf  100, 500 mg/kg and 1 g/kg of body wt of ethanol extract | Positive control: IP dexamethasone 0.75 mg and negative control: IP distilled water 0.5 mL;  Positive control: P.O. diclofenac and negative control: P.O. drinking water;  Positive control: P.O. diclofenac sodium 40 mg/kg body wt and negative control: P.O. distilled water for carrageenan induced paw edema and positive control: indomethacin 5 mg/kg body wt for cotton pallet induced granuloma test | *A indica* aqueous extract inhibited formalin induced paw edema significantly; 500 mg/kg of ethanol extract inhibited paw edema significantly where maximum inhibition exerted at 3 h after injection with carrageenan;  1 g/kg of ethanol extract inhibited paw edema significantly in the time of fours after injection with carrageenan where maximum inhibition exerted at 3 h and significantly reduced the weight of cotton pellets | Not determined;  **Acute toxicity test and brine shrimp lethality test:** In acute toxicity test, the extract was administered in graded doses of 250 and 500 mg/kg body wt as single oral dose and was found safe since no mortality or significant changes in behavior, breathing, cutaneous effects, sensorynervous system responses or gastrointestinal effects was observed in any group after 24 h of administration. On the other hand, a LC_50_ value of 30.20 μg/mL was obtained with the ethanol extract in brine shrimp lethality test;  **Acute toxicity test:** The extract was administered as a single oral dose of 4 g/kg body wt, observed closely for first 24 h and also next 10 days for delayed toxic effect. No mortality or toxicity was noted | ([Mosaddek and Rashid, 2008](#_ENREF_201))  ([Emran et al., 2015](#_ENREF_81))  ([Mahabub-Uz-Zaman et al., 2009](#_ENREF_190)) |
| *Butea monosperma* (Lam.) Taub. (Fabaceae)  - Parrot tree or Bengal kino tree (Polash or kingsuk)  ([Ghani, 2003](#_ENREF_95)) | **Leaf:** diuretic; used to cure boils,pimples, tumor, worm, hemorrhages and piles. **Flower:** emmenagogue; decoction is used in diarrhea. **Bark:** astringent and aphrodisiac; used to treat tumor, bleeding piles and ulcer; decoction is useful in catarrh, cold, cough, fever, hemorrhages and menstrual disorders. **Gum:** astringent; useful in bruises, erysipelatous inflammation and ringworm; decoction or tincture is used in acid indigestion, diarrhea and dysentery. **Seed:** laxative and anthelmintic; seed paste is useful in ringworm ([Ghani, 2003](#_ENREF_95)) | *In vivo,* cotton pellet induced granuloma and croton oil induced ear edema;  *In vitro,* human RBC membrane stabilization assay | Root  P.O. 600 and 800 mg/kg body wt of ethanol extract;  Leaf  2 mg/mL of methanol ex-tract and its petroleum ether, carbon tetrachloride, chloroform and aqueous soluble fraction | Positive control: P.O. diclofenac-sodium 100 mg/kg body wt;  Positive control: acetyl salicylic acid 0.10 mg/mL | 600 and 800 mg/kg body wt of ethanol extract reduced the weight of cotton pellets and inhibited croton oil induced ear edema;  2 mg/mL of methanol extract and its fractions significantly inhibited hemolysis induced by hypotonic solution and heat | **Acute toxicity test**: The extract was found to be safe up to 4,000 mg/kg body wt P.O.;  Not determined | ([Arefin et al., 2015](#_ENREF_23))  ([Uddin et al., 2018](#_ENREF_287)) |
| *Clerodendrum infortunatum* L. or *Clerodendrum viscosum* Vent. (Lamiaceae)  - Hill glory bower (Bhant or vita or gatupata) | **Leaf:** juice is anthelmintic, emetic, laxative and cholagogue; infusion is bitter tonic, antiperiodic in malaria and used in the treatment of chest complaints with coughs and asthma. **Root:** anthelmintic and antifungal  ([Ghani, 2003](#_ENREF_95)) | *In vivo*, carrageenan induced paw edema;  *In vitro,* human RBC membrane stabilization assay | Whole plant P.O. 150 mg and 300 mg/kg body wt of methanol extract; Leaf  200 mg and 400 mg/kg body wt of methanol extract;  Root  2 mg/mL of each of methanol extract and its ethyl acetate, petroleum ether, chloroform and aqueous soluble fraction | Positive control: P.O. phenylbutazone 100 mg/kg body wt;  Positive control: P.O. phenylbutazone 100 mg/kg body wt and negative control: P.O. 1% Tween 80 solution in water 10 mL/kg of body wt;  Positive control: acetyl salicylic acid 0.10 mg/mL | 150 mg and 300 mg/kg body wt of methanol extract inhibited paw edema significantly in the time of 3 h after injection with carrageenan;  200 mg and 400 mg/kg body wt of methanol extract inhibited paw edema significantly in the time of 5 h after injection with carrageenan;  2 mg/mL of each extract significantly inhibited lysis of RBC membrane induced by hypotonic solution whereas 2 mg/mL of ethyl acetate soluble fraction significantly inhibited heat induced hemolysis | Not determined;  Not determined;  Not determined | ([Khatry et al., 2005](#_ENREF_164))  ([Ahmed et al., 2007](#_ENREF_3))  ([Sabikunnahar et al., 2016](#_ENREF_241)) |
| *Coccinia. grandis* (L.) Voigt or *Coccinia grandis* L. or *C. indica* (Cucurbitaceae)  - Ivy gourd (Telakucha) | **Leaf:** used in the treatment of diabetes; externally used in skin eruption.  Infusion of different plant part is used in anorexia, epilepsy, catarrh, asthma, fever, dropsy and gonorrhea ([Ghani, 2003](#_ENREF_95)) | *In vitro,* human RBC membrane stabilization assay | Leaf  1 mg/mL of ethanol extract;  1 mg/mL of each of ethanol extract and its petroleum ether, hexane, carbon tetrachloride and aqueous fraction | Positive control: acetyl salicylic acid 0.10 mg/mL;  Positive control: acetyl salicylic acid 0.10 mg/mL | 1 mg/mL of extract significantly inhibited lysis of human RBC membrane induced by hypotonic solution and heat;  1 mg/mL of extract and fractions significantly inhibited hemolysis induced by hypotonic solution and heat | **Brine shrimp lethality test:** A LC_50_ value of 13.69 μg/mL was obtained with the ethanol extract;  Not determined | ([Laboni et al., 2017](#_ENREF_177))  ([Sultana et al., 2018](#_ENREF_275)) |
| *Crinum asiaticum* L. (Amaryllidaceae) - Poison bulb (Bara kanur)  ([Ghani, 2003](#_ENREF_95)) | **Leaf:** used in earache; with oil used to treat whitlows and other inflammatory conditions of toes, fingers and joints. **Leaf and root:** used as emetic, diaphoretic and purgative. **Bulb:** laxative, expectorant, tonic and rubefacient in rheumatism **(**[Ghani, 2003](#_ENREF_95)**)** | *In vivo*, carrageenan induced paw edema | Bulb  P.O. 1, 1.5 and 2 gm/kg body wt of methanol extract;  Leaf  P.O. 1 and 2 gm/kg body wt of ethanol extract;  Leaf  P.O. 1 and 2 gm/kg body wt of ethanol extract | Positive control: P.O. diclofenac sodium 40 mg/kg body wt and negative control: P.O. distilled water;  Positive control: P.O. diclofenac sodium 10 mg/kg body wt;  Positive control: P.O diclofenac sodium 10 mg/kg body wt and negative control: P.O. distilled water | C. *asiaticum* methanol extract dose dependently inhibited paw edema in the time of 4 h after injection with carrageenan;  2 gm/kg of ethanol extract inhibited paw edema significantly in the time of 4 h after injection with carrageenan;  2 gm/kg of ethanol extract inhibited paw edema significantly in the time of 4 h after injection with carrageenan | Not determined;  Not determined;  **Acute toxicity test:** The extract was administered in graded doses of 0.5, 1.0, 2.0, 3.0 and 4 g/kg body wt as single oral dose, observed closely for first 24 h and also next 14 days for delayed toxic effect. No mortality or toxicity was noted | ([Rahman et al., 2011a](#_ENREF_227))  ([Uddin et al., 2012](#_ENREF_290))  ([Rahman et al., 2013](#_ENREF_226)) |
| *Cyanthillium cinereum* (L.) H.Rob. or  *Vernonia cinerea* (L.) Less. (Asteraceae) - Little ironweed  (Sahadevi)  ([Guha et al., 2011](#_ENREF_103)) | **Flowers:** are used in the treatment of conjunctivitis, arthritis and rheumatism. **Stem and bark:** are used to heal cuts. **Root:** infusion is used as antidote for scorpion sting and snake bite. **Seed:** anthelminticand are used as alterative in leprosy and in chronic skin disease. **Whole plant:** decoction is used to treat fever, spasms of the urinary bladder and strangury and in malaria with quinine ([Guha et al., 2011](#_ENREF_103)) | *In vitro,* protein (albumin) denaturation assay;  *In vitro,* human RBC membrane stabilization assay | Whole plant  1 mg/mL of methanol extract;  100, 200 and 400 μg/mL of methanol extract | Positive control: acetyl salicylic acid 0.1 mg/mL;  Positive control: acetyl salicylic acid 100 μg/mL and negative control: methanol | 1 mg/mL of extract significantly inhibited both heat and hypotonicity induced hemolysis;  *V. cinerea* methanol extract significantly inhibited heat induced protein denaturation in a dose dependent manner | **Brine shrimp lethality test:** No LC_50_ value was obtained with the methanol extract since no mortality observed in the assay;  Not determined | ([Kawsar et al., 2011](#_ENREF_159))  ([Bashar et al., 2014](#_ENREF_36)) |
| *Cyanthillium patulum* (Dryand. ex Dryand.) H.Rob. or  *Vernonia patula* (Dryand.) Merr (Asteraceae)  - Pubple fleabane  (Kukshim, or kukshima or kukursunga)  ([Ghani, 2003](#_ENREF_95)) | **Leaf:** fresh juice is given in the treatment of amoebiasis, as a poultice in humid herpes, eczema, ringworm, guinea worm and elephantiasis. **Flower:** used in conjunctivitis, fever and rheumatism. **Root:** anthelmintic; decoction is given in diarrhea, stomachache, dropsy, cough and colic. **Seed:** alterative, anthelmintic and alexipharmic; useful in cough, flatulence, intestinal colic, dysuria, leukoderma, psoriasis and other skin diseases  ([Ghani, 2003](#_ENREF_95)) | *In vivo*, carrageenan and histamine induced paw edema;  *In vitro,* human RBC membrane stabilization assay | Aerial parts P.O. 100, 200 and 400 mg/kg body wt of ethanol extract;  Leaf and stem  1 mg/mL of ethanol extract of both leaf and stem | Positive control: P.O. indomethacin 10 mg/kg body wt and negative control: P.O. 1 % Tween 80 in normal  saline 10 mL/kg body wt;  Positive control: acetyl sali-cylic acid 0.10 mg/mL | 400 mg/kg of *V. patula* extract significantly inhibited paw edema in 5 h after injection with carrageenan as well as with histamine;  1 mg/mL of ethanol extract of both leaf and stem significantly protected the lysis of human erythrocyte membrane induced by hypotonic solution | **Acute toxicity test:** The extract was administered in graded doses of 200, 400, 800, 1,600 and 3,200 mg/kg body wt as single oral dose and was found safe up to 3,200 mg/kg since no mortality or toxic reaction was recorded in any group after 72 h of administration;  Not determined | ([Hira et al., 2013](#_ENREF_121))  ([Sufian et al., 2017](#_ENREF_273)) |
| *Eclipta prostrata* (L.) L. *or E. alba* (L.) Hassk. (Asteraceae) - False daisy (Kesuti, keshraj bhimraj or kalokeshi)  ([Ghani, 2003](#_ENREF_95)) | **Leaf:** used in sores, hemorrhages, acidity, hurt burn and elephantiasis. **Pulp:** pulp roasted in ghee is used in the treatment of wounds and burns.  This plant act as tonic, stomachic, antipyretic, anthelmintic and expectorant and is useful in the treatment of asthma, inflammation, hernia, eye disease, skin disease, leukoderma, itching, anemia and bronchitis; juice of this plant promotes hair growth and improves hair color ([Ghani, 2003](#_ENREF_95)) | *In vivo*, carrageenan induced paw edema;  *In vitro*, human RBC membrane stabilization assay | Aerial parts P.O. 200 and 400 mg/kg body wt of each of methanol extract and its dichloromethane and ethyl acetate fraction;  Leaf  2 mg/mL of each of ethanol extract and its hexane, chloroform and aqueous soluble fraction | Positive control: P.O. indomethacin 10 mg/kg body wt and negative control:  distilled water 3 mL/kg;  Positive control: acetyl salicylic acid 0.10 mg/mL | 200 and 400 mg/kg body wt of each of sample inhibited paw edema significantly in a dose dependent manner in the time of 3 h after injection with carrageenan;  2 mg/mL of each of ethanol extract and its hexane and chloroform fraction significantly inhibited heat and hypotonicity induced hemolysis | **Acute toxicity test:** The extract and fractions were administered in graded doses of 250, 500, 1,000 and 1,500 mg/kg body wt as single oral dose and were found safe up to 1,500 mg/kg since no mortality was recorded in any group after 48 h of administration, but little behavioral changes, locomotor ataxia, diarrhea and weight loss were observed;  Not determined | ([Hossain et al., 2011](#_ENREF_131))  ([Uddin et al., 2015](#_ENREF_288)) |
| *Euphorbia hirta* L. (Euphorbiaceae) - Australian asthma weed or snakeweed  (Bara-keru, bara dudhi or ghaopata)  ([Ghani, 2003](#_ENREF_95)) | This plant is useful in the treatment of cough, bowel complaints, worms of children; juice of this plant possesses tonic, antiasthmatic and febrifuge properties and is useful against diarrhea and dysentery; decoction of this plant is used to treat bronchial asthma, chronic bronchitis and other respiratory tract infections ([Ghani, 2003](#_ENREF_95)) | *In vivo,* xylene induced ear edema and cotton pallet induced granuloma | Leaf  P.O. 200, 400 and 800 mg/kg body wt of methanol extract | Positive control: P.O. diclofenac-sodium 100 mg/kg body wt and negative control: distilled  water 10 mL/kg body wt | *E. hirta* methanol extract significantly inhibited xylene induced ear edema as well as reduced the weight of cotton pellets in a dose dependent manner | **Acute toxicity test:** The extract was administered in graded doses of 100, 200, 400, 800, 1,600 and 3,200 mg/kg body wt as single oral dose and was found safe up to 3,200 mg/kg since no mortality or toxic reaction was recorded in any group after 24 h of administration | ([Rahman et al., 2019](#_ENREF_229)) |
| *Flemingia stricta* Roxb. (Fabaceae) - (Charchara or uskura) | **Root:** fresh juice isused to cure asthma  ([Uddin, 2006](#_ENREF_293)) | *In vitro,* human RBC membrane stabilization assay and protein (albumin) denaturation assay | Leaf  31.25, 62.5, 125, 250, 500, 1,000 μg/mL of methanol extract | Positive control: diclofenac sodium 31.25, 62.5, 125, 250, 500, 1,000 μg/mL | The extract dose dependently inhibited hypotonicity induced hemolysis as well as heat induced protein denaturation | Not determined | ([Biozid et al., 2015](#_ENREF_47)) |
| *Glycosmis pentaphylla* (Retz.) DC*.* or *Glycosmis pentaphylla* (Retz.) DC*.* or *G. arborea* (Rutaceae)  - Jamaica mandarin orange or orange berry  (Ashsaora, kawatuti,  Matmati or ban jamir) | **Leaf:** infusion is used in fever, liver complaints, cough, rheumatism, anemia and jaundice and also used for anthelmintic property. **Fruit and root :** used in fever ([Ghani, 2003](#_ENREF_95)) | *In vitro,* human RBC membrane stabilization assay | Leaf and stem  2 mg/mL of methanol extract of each of leaf and stem and their petroleum ether, carbon tetrachloride, chloroform and aqueous soluble fraction;  Leaf  62.5, 125, 250, 500 and 1,000 μg/mL of methanol extract;  Part not found  0.5 and 1 mg/mL of ethanol extract | Positive control: acetyl salicylic acid 0.10 mg/mL;  Positive control: diclofenac 62.5, 125, 250, 500 and 1,000 μg/mL;  Positive control: acetyl salicylic acid 0.10 mg/mL | 2 mg/mL of extracts and fractions inhibited lysis of human RBC membrane induced by hypotonic solution and heat in varying degree;  *G. pentaphylla* extract significantly inhibited hemolysis induced by hypotonic solution in a dose dependent manner;  0.5 and 1 mg/mL of extract moderately inhibited hemolysis induced by heat | Not determined;  Not determined;  Not determined | ([Hossain et al., 2012c](#_ENREF_132))  ([Ansari et al., 2015](#_ENREF_21))  ([Chowdhury et al., 2015](#_ENREF_59)) |
| *Gynura nepalensis* DC. (Asteraceae) - Philippine Ashitaba (Sidirebaishak, titlong or baishak)  ([Uddin, 2006](#_ENREF_293)) | **Leaf:** paste is used externally to treat boils, edema, eczema, tumor, headache, food poisoning and dysmenorrhea; water vapor is effective in fever; pills prepared from leaves with honey is given in hyper acidity; in hysteria pills prepared from leaves are given and hot water bath with leaf extract is advised;pills prepared from leaves and stem is given in tape worm infestation and in constipation; paste prepared from leaf and stem is used in ureterolithiasis. **Root:** fresh juice is used to treat body and chest pain, fever, tumor and mental disorder; pills prepared from root are given in headache and food poisoning. **Leaf and root:** fresh juice is used in epilepsy; pills made from leaf and root is given in paraplegia and at the same time paste from leaf and root is applied externally  ([Uddin, 2006](#_ENREF_293)) | *In vitro,* protein (albumin) denaturation and human RBC membrane stabilization assay | Leaf  0.12, 0.18, 0.24, 0.30 and 0.36% (v/v) aqueous extract | Positive control: acetyl salicylic acid 100 μg/mL and negative control: distilled water | The extract dose dependently inhibited heat induced protein denaturation as well as heat induced hemolysis | Not determined | ([Aktar et al., 2019](#_ENREF_9)) |
| *Heliotropium indicum* L. (Boraginacea) - Helioprope  (Hatisur or hatisura)  ([Ghani, 2003](#_ENREF_95)) | **Leaf:** juice is useful to treat eye diseases and used locally to ulcer, sore, wound, gum boils, skin infection and insect stings. **Leaf and young shoot:** decoction is used in urticaria, ringworm, rheumatism, gonorrhea, pharyngitis and tonsillitis. **Root:** aphrodisiac and is used to cure night blindness  ([Ghani, 2003](#_ENREF_95)) | *In vitro,* human RBC membrane stabilization assay | Whole plant  1 mg/mL of each of methanol extract and its hexane, carbon tetrachloride, chloroform and aqueous soluble fraction;  Leaf  2 mg/mL of each of ethanol extract and its petroleum ether, carbon tetrachloride and chloroform fraction;  Leaf and root  2 mg/mL of methanol extract from each of leaf and root and their *n*-hexane, carbon tetrachloride, chloroform and aqueous soluble fraction | Positive control: acetyl salicylic acid 0.10 mg/mL;  Positive control: acetyl salicylic acid 0.10 mg/mL;  Positive control: acetyl salicylic acid 0.10 mg/mL | 1 mg/mL of extract and fractions inhibited lysis of human RBC membrane induced by hypotonic solution and heat in varying degree;  2 mg/mL of extract and fractions inhibited lysis of human RBC membrane induced by hypotonic solution and heat in varying degree;  2 mg/mL of *n*-hexane and chloroform soluble fractions of both leaf and root significantly inhibited lysis of human RBC membrane induced by hypotonic solution and heat | **Brine shrimp lethality test:** LC_50_ values of 2.57, 3.91, 9.69, 31.44 and 24.98 μg/mL were obtained with methanol extract and its hexane, carbon tetrachloride, chloroform and aqueous soluble fractions respectively;  Not determined;  Not determined | ([Mourin et al., 2013](#_ENREF_202))  ([Samira et al., 2016](#_ENREF_245))  ([Hamiduzzaman et al., 2017](#_ENREF_111)) |
| *Heritiera fomes* Buch.-Ham. (Malvaceae) - (Sundori)  ([Uddin, 2019](#_ENREF_292)) | **Bark:** paste is applied to eczema, abscesses, boils and scabies affected part of the body  Other uses of the plant includes jaundice and hepatitis ([Uddin, 2019](#_ENREF_292)) | *In vitro,*  human RBC membrane stabilization assay and *in vivo*, carrageenan induced paw edema | Leaf and bark  For *in vitro* assay, 50, 100, 200, 400 and 800 μg/mL of ethanol extract from each of leaf and bark and for *in vivo* assay, P.O. 1, 5, 10, 20 and 40 mg/kg body wt of ethanol extract from each of leaf and bark | For *in vitro* assay, positive control: diclofenac sodium 012.5, 25, 50, 100 and  200 μg/mL and for *in vivo* assay, positive control: P.O. indomethacin 10 mg/kg body wt, negative control: normal saline | Both bark and leaf extracts dose dependently inhibited hemolysis induced by hypotonic solution and heat and inhibited paw edema in the time of 4 h after injection with carrageenan | Not determined | ([Islam et al., 2020a](#_ENREF_139)) |
| *Lantana camara* L. (Verbenaceae) - Lantana  (Chotra) | **Leaf and shoot:** decoction is used to treat tetanus, rheumatism, malaria and atony of abdominal viscera.  This plant is also known for its vulnerary, diaphoretic, carminative and antiseptic properties ([Ghani, 2003](#_ENREF_95)) | *In vitro,* protein (albumin)  denaturation assay and human RBC membrane stabilization assay | Whole plant 125, 250 and 500 μg/mL of methanol extract | Positive control: acetyl salicylic acid 125, 250 and 500 μg/mL | The extract significantly inhibited heat induced protein denaturation as well as hypotonicity induced hemolysis in a dose dependent manner | Not determined | ([Ripon et al., 2016](#_ENREF_240)) |
| *Lawsonia inermis* L. (Lythraceae) - Henna  (Mehandi) | **Leaf:** paste is used externally to skin diseases, dandruff, burning of feet, headache and as emollient poultice; decoction is used as astringent gargle in sore throat and to treat diarrhea and dysentery. **Bark:** decoction is used externally to burns and scalds and internally to treat jaundice, spleen enlargement, leprosy, obstinate skin diseases. **Flower:** soporific and has cooling properties ([Ghani, 2003](#_ENREF_95)) | *In vivo*, acetic acid induced writhing test;  Carrageenan induced paw edema | Leaf  P.O. 200, 300, 400 and 500 mg/kg body wt of methanol extract;  Bark  P.O. 300 and 500 mg/kg body wt of methanol extract | Positive control: P.O. diclofenac sodium 20 mg/kg body wt;  Positive control: IP ibuprofen 10 mg/kg body wt and negative control: P.O. normal saline 1 mL/kg body wt | The extract significantly reduced acetic acid induced writhing in a dose dependent manner;  300 and 500 mg/kg of extract inhibited paw edema significantly in the time of 4 h after injection with carrageenan | Not determined;  Not determined | ([Imam et al., 2013](#_ENREF_137))  ([Nesa et al., 2014](#_ENREF_208)) |
| *Leea macrophylla* Roxb. ex Hornem. (Leeaceae) - Elephant ears (Dholsamudra) | **Leaf:** paste is applied externally to boils, gout and tumor. **Root:** paste prepared from roasted root is applied externally on affected throat as a treatment of goiter; fresh juice is useful in tetanus. **Stem and root:** fresh juice is used in rheumatic pain ([Uddin, 2006](#_ENREF_293)) | *In vitro,* protein (albumin) denaturation and human RBC membrane stabilization assay | Leaf  500 μg/mL of ethanol extract | Positive control: acetyl salicylic acid 0.1% | 500 μg/mL of extract significantly inhibited heat induced protein denaturation and moderately inhibited hypotonicity induced hemolysis | Not determined | ([Faruq et al., 2014](#_ENREF_87)) |
| *Mallotus repandus* (Willd.) Müll.Arg. (Euphorbiaceae) - Climbing liana (Gunti, jhante, or bon natai) | **Leaf:** used for anti-inflammatory properties.  Other medicinal uses of this plant recorded are in the treatment of fever, rheumatic arthritis, snake bite, hepatitis, liver cirrhosis, ulcer, tumor, muscle pain and as insecticide  ([Hasan et al., 2014](#_ENREF_115); [Hasan et al., 2018](#_ENREF_116)) | *In vivo,* xylene induced ear edema and cotton pallet induced granuloma;  Xylene induced  ear edema, carrageenan induced paw edema and cotton pellet induced granuloma | Leaf  P.O. 500, 1,000, and 2,000 mg/kg body wt of methanol extract;  Stem  P.O. 500, 1,000, and 2,000 mg/kg body wt of methanol extract | Positive control: P.O. diclofenac sodium 100 mg/kg body wt and negative control: P.O. 1% Tween 80 in water 10 mL/kg;  Positive control: P.O. diclofenac sodium 100 mg/kg body wt for ear and paw edema model and ibuprofen 100 mg/kg for granuloma model | The extract inhibited ear edema and granuloma formation in a dose dependent manner where highest significance exerted with 2,000 mg/kg;  The extract inhibited ear edema, paw edema and granuloma formation in a dose dependent manner where highest significance exerted with 2,000 mg/kg | **Acute toxicity test:** The extract was administered in graded doses of 250, 500, 1,000 2,000 and 4,000 mg/kg body wt using stomach tube and was found safe up to 4,000 mg/kg since no mortality or sign of restlessness, respiratory distress, general irritation, coma, or convulsion was recorded in any group;  **Acute toxicity test:** The extract was administered in graded doses of 1,000, 2,000, 3,000 and 4,000 mg/kg body wt using stomach tube, observed closely for first 24 h and also next 3 days. and was found safe up to 4,000 mg/kg since no mortality or sign of restlessness, respiratory distress, general irritation, coma, or convulsion was recorded in any group | ([Hasan et al., 2014](#_ENREF_115))  ([Hasan et al., 2018](#_ENREF_116)) |
| *Mangifera indica* L. (Anacardiaceae) - Mango  (Aam gachh or amro)  ([Ghani, 2003](#_ENREF_95)) | **Leaf:** immature leaf extract is anthelmintic; decoction is used in fever, diarrhea and toothache. **Fruit:** green fruits are astringent and stimulant tonic and are given in debility of stomach, ophthalmia and eruptions; ripe fruits are nutritious and are laxative. **Bark:** is astringent and used in diphtheria and rheumatism; resinous juice of the bark is used to treat syphilis, scabies and cutaneous infections. **Bark and seed kernel:** astringent and useful in hemorrhages and diarrhea. **Kernel:** anthelmintic and efficacious in bleeding piles and menorrhagia; juice can stop nasal bleeding if sniffed. **Flower:** dried flowers have astringent property and are used in diarrhea, chronic dysentery and catarrh of the bladder and gleet ([Ghani, 2003](#_ENREF_95)) | *In vivo*, carrageenan induced paw edema | Leaf  P.O. 2 g/kg body wt of ethanol extract;  P.O. 2 g/kg body wt of ethanol extract | Positive control: P.O. diclofenac sodium 40 mg/kg body wt and negative control:  distilled water;  Positive control: P.O. diclofenac sodium 40 mg/kg body wt and negative control:  distilled water; | Significant inhibition of paw edema observed at 2-3 h after injection with carrageenan;  2 g/kg extract inhibited paw edema in the time of 4 h after injection with carrageenan; | Not determined;  Not determined | ([Islam et al., 2010](#_ENREF_138))  ([Hassan et al., 2013](#_ENREF_118)) |
| *Manilkara zapota* (L.) P.Royen (Sapotaceae) -Sapoti, chickle gum, American bully (Sopheda or sobera) | **Leaf:** used to treat cold, cough and diarrhea. **Bark:** used in fever, pain, gastrointestinal disorders and in inflammatory conditions ([Ganguly et al., 2013](#_ENREF_93)) | *In vivo,* carrageenan and histamine induced paw edema;  Carrageenan induced paw edema | Bark  P.O. 200 and 400 mg/kg body wt of methanol extract;  Leaf  P.O. 300 mg/kg body wt of ethanol extract and its petroleum ether, ethyl acetate and carbon tetrachloride fraction | Positive control: P.O. indomethacin 10 mg/kg body wt and negative control: P.O. % Tween 80 in normal saline 2 mL/kg;  Positive control: P.O. diclofenac sodium 100 mg/kg body wt and negative control: P.O. 1% Tween 80 in normal saline 10 mL/kg | 400 mg/kg of extract inhibited paw edema significantly in the time of 4 h after injection with carrageenan and histamine;  300 mg/kg of ethanol and ethyl acetate extract inhibited paw edema significantly in the time of 6 h after injection with carrageenan | **Acute toxicity test:** The extract was administered in graded doses of 200, 400, 800, 1,600 and 3,200 mg/kg body wt as single oral dose and was found safe up to 3,200 mg/kg since no mortality or toxic reaction was recorded in any group after 48 h of administration;  **Acute toxicity test:** The extract and fractions were administered in graded doses of 200, 400, 800, 1,600 and 3,200 mg/kg body wt as single oral dose and was found safe up to 3,200 mg/kg since no mortality or toxic reaction was recorded in any group after 48 h of administration | ([Hossain et al., 2012a](#_ENREF_127))  ([Ganguly et al., 2013](#_ENREF_93)) |
| *Microcos paniculata* L. (Tiliaceae) - (Kathgua, fattashi, patka, assor gular, pesondi or pichunti)  Status: ambiguous | **Leaf:** paste is applied on fractured bone in case of bone fracture and on forehead in case of headache; fresh extract is used externally in dysmenorrhea; pills prepared from leaves are given in rheumatic arthritis. **Fruit:** paste is applied externally on boils. **Shoot:** fresh extract from young shoot is used to treat bone sprain. **Root:** fresh juice is given in paratyphoid. **Leaf, bark and root:** used in the treatment of tumor ([Uddin, 2006](#_ENREF_293)) | *In vitro,* proteinase inhibitory assay and  *in vivo,* xylene induced ear edema and cotton pallet induced granuloma;  *In vitro,* proteinase-inhibitory assay;  *In vitro,* human RBC membrane stabilization assay;  *In vivo,* xylene induced ear edema and cotton pallet induced granuloma | Bark and fruit  For *in vitro* assay, 50–250 μg/mL and for *in vivo* assays, P.O. 200 and 400 mg/kg body wt of methanol extract from each of bark and fruit;  Fruit  50–250 μg/mL of aqueous extract;  Stem bark  2-10 mg/mL of methanol extract;  Bark  P.O. 200 and 400 mg/kg body wt of hydro-methanol and petroleum-benzene extract | Positive control: acetyl salicylic acid 50–250 μg/mL and P.O. diclofenac sodium 100 mg/kg body wt for *in vitro* and *in vivo* assays respectively;  Positive control: acetyl salicylic acid 50–250 μg/mL;  Positive control: acetyl salicylic acid 0.1 mg/mL;  P.O. diclofenac sodium 100 mg/kg body wt | *M. paniculata* bark extract showed maximum antiproteinase activity with IC_50_ value of 61.31 μg/mL and maximum inhibition of ear edema and granuloma formation also obtained with bark extract 400 mg/kg;  The aqueous fruit extract showed an antiproteinase activity with IC_50_ value of 285.47 μg/mL;  8 and 10 mg/mL of extracts significantly inhibited hemolysis induced by hypotonic solution;  400 mg/kg of both extract showed maximum inhibition of ear edema and granuloma formation | **Acute toxicity test:** Both bark and fruit extracts were administered in graded doses of 100, 250, 500, 1,000, 2,000, 3,000 and 4,000 mg/kg body wt as single oral dose, observed closely for first 5-6 h and also next 2 weeks. No mortality or toxicity was noted;  Not determined;  Not determined;  Not determined | ([Aziz, 2015](#_ENREF_28))  ([Aziz et al., 2015](#_ENREF_30))  ([Al-Amin Sarker et al., 2016](#_ENREF_11))  ([Aziz et al., 2018](#_ENREF_29)) |
| *Mussaenda roxburghii* Hook.f. (Rubiaceae) *- East Himalayan Mussaenda* (Chauri-chaonri gach, sildaura or supaila) | **Leaf:** paste is applied on forehead to cure headache, on tumor infected part of the body to cure tumor, on breast to cure breast pain, on cuts to stop bleeding, and is useful in skin disease and snake bite; pills prepared from leaf is given in hyperacidity, rheumatism and cirrhosis; decoction is given in paralysis. **Leaf and flower:** paste is applied externally on the navel area to relieve abdominal pain. **Leaf and stem:** extract used in edema. **Root:** fresh juice is given in gout, pyorrhea and food poisoning; pills made from root is given in epilepsy ([Uddin, 2006](#_ENREF_293)) | *In vitro,* protein (albumin) denaturation and human RBC membrane stabilization assay;  *In vitro,* protein (albumin) denaturation assay | Leaf  31.25, 62.5, 125, 250, 500, 1,000 μg/mL of methanol extract;  Root  100, 200 and 400 μg/mL of each of methanol extract and its dichloromethane and ethyl acetate fraction | Positive control: diclofenac sodium 31.25, 62.5, 125, 250, 500, 1,000 μg/mL;  Positive control: diclofenac sodium 100, 200 and 400 μg/mL | *M. roxburghii* extract significantly inhibited heat induced protein denaturation in a concentration dependent manner;  The crude extract and fraction inhibited heat induced protein denaturation in a concentration dependent manner | Not determined;  **Brine shrimp**  **lethality test:** LC_50_ values of 52.38, 50.20 and 32.46 μg/mL were obtained with methanol extract and its dichloromethane and ethyl acetate fractions respectively | ([Chowdury et al., 2015](#_ENREF_60))  ([Sumi et al., 2015](#_ENREF_276)) |
| *Oroxylum indicum* (L.) Kurz (Bignoniaceae) - Broken bones or midnight horror (Sonapatha, dashmula, or shyonaka) | **Leaf:** emollient. **Bark:** powder and infusion is diaphoretic and tonic and are useful in rheumatism. **Stem:** useful in scorpion sting. **Root:** useful in dropsy and wound healing; root bark is astringent, tonic, stomachic, anodyne and sudorific and is useful in diarrhea and dysentery. **Seed:** purgative ([Ghani, 2003](#_ENREF_95)) | *In vitro,* human RBC membrane stabilization assay;  *In vivo*, carrageenan induced paw edema | Bark and leaf  2 mg/mL of methanol extract from each of bark and leaf and their petroleum ether, carbon tetrachloride, chloroform and aqueous soluble fraction;  Stem bark  P.O. 100, 200 and 400 mg/kg body wt of methanol extract | Positive control: acetyl salicylic acid 0.10 mg/mL;  Positive control: IP ibuprofen 20 mg/kg body wt | 2 mg/mL of each extract inhibited lysis of human RBC membrane induced by hypotonic solution in varying percentage where maximum inhibition obtained with methanol extract and aqueous soluble fraction;  100, 200 and 400 mg/kg extract inhibited paw edema significantly at 6 h after injection with carrageenan | **Brine shrimp lethality test:** LC_50_ values of 17.08, 1.81, 16.11, 15.03 and 20.93 μg/mL were obtained with bark methanol extract and its petroleum ether, carbon tetrachloride, chloroform and aqueous soluble fractions respectively whereas, LC_50_ values of 12.83, 22.27, 6.54, 8.40 and 12.03 μg/mL were obtained with leaf methanol extract and its petroleum ether, carbon tetrachloride, chloroform and aqueous soluble fractions respectively;  Not determined | ([Chakma et al., 2013](#_ENREF_53))  ([Begum et al., 2019](#_ENREF_41)) |
| *Phrynium imbricatum* Roxb. (Marantaceae) - (Khedom gas) | **Root:** paste is used to treat rheumatism ([Uddin, 2019](#_ENREF_292)) | *In vitro,* protein (albumin) denaturation and human RBC membrane stabilization assay | Leaf  25, 50, 100, 200 and 400 μg/mL of ethanol extract | Positive control: diclofenac sodium 25, 50, 100, 200 and 400 μg/mL | The extract inhibited heat induced protein denaturation as well as hypotonicity induced hemolysis in a dose dependent manner | Not determined | ([Hossain et al., 2015](#_ENREF_130)) |
| *Phyllodium pulchellum* (L.) Desv. or  *Desmodium pulchellum* (L.) Benth. (Fabaceae) - (Juta salpani)  ([Ghani, 2003](#_ENREF_95)) | **Leaf:** used to treat ulcer. **Bark:** decoction is used in diarrhea, eye afflictions, malaria, swelling and rheumatism ([Noor et al., 2013](#_ENREF_212)) | *In vivo*, carrageenan induced paw edema and cotton pallet induced granuloma | P.O. 100, 200 and 400 mg/kg body wt of ethanol extract | Positive control: P.O. diclofenac sodium 5 mg/kg body wt and negative control: normal saline  10 mL/kg body wt | 400 mg/kg of ethanol extract significantly inhibited paw edema in the time of 3 h after injection with carrageenan and reduced the weight of cotton pellets | **Acute toxicity test:** The extract was found to be safe up to 4,000 mg/kg body wt P.O. | ([Noor et al., 2013](#_ENREF_212)) |
| *Piper retrofractum* Vahl  or *Piper chaba* Hunter (Piperaceae) - Javanese long pepper (Choi) | **Fruit:** stimulant, carminative, pungent, thermogenic, anthelmintic and expectorant; they are useful in asthma, bronchitis, fever, inflammation, piles, pain in the abdomen and anus, and to enhance taste and appetite. **Stem:** useful to relieve pain after child birth, rheumatic pain, and diarrhea. **Root:** alexiteric; useful in asthma, bronchitis and consumption ([Taufiq-Ur-Rahman et al., 2005](#_ENREF_280)) | *In vivo*, carrageenan induced paw edema;  *In vivo*,  cotton pallet induced granuloma;  *In vivo*, carrageenan induced paw edema;  *In vitro,* human RBC membrane stabilization and protein (albumin) denaturation assay | Stem bark P.O. 125, 250 and 500 mg/kg body wt of methanol extract;  P.O. 125 and 250 mg/kg body wt of methanol extract;  P.O. 125 and 250 mg/kg body wt of methanol extract;  Root  100, 200, 300, 400 and 500 μg/mL of ethanol extract | Positive control: P.O. phenylbutazone 100 mg/kg body wt and negative control: P.O. normal saline;  Positive control: P.O. aspirin 100 mg/kg body wt, hydrocortisone 2 mg/kg body wt subcutaneously and negative control: P.O. normal saline;  Positive control: P.O. aspirin 100 mg/kg body wt, hydrocortisone 2 mg/kg body wt subcutaneously and negative control: P.O. normal saline;  Positive control: acetyl salicylic acid 100, 200, 300, 400 and 500 μg/mL | The extract inhibited paw edema significantly in a dose dependent manner after injection with carrageenan;  The extract inhibited granuloma formation significantly in a dose dependent manner;  The extract inhibited paw edema significantly in a dose dependent manner in the time of 3 h after injection with carrageenan;  *P. chaba* significantly inhibited heat and hypotonicity induced hemolysis as well as heat induced protein denaturation in a dose dependent manner | Not determined;  Not determined;  Not determined;  Not determined | ([Taufiq-Ur-Rahman et al., 2005](#_ENREF_280))  ([Begum et al., 2008](#_ENREF_40))  ([Begum et al., 2012](#_ENREF_39))  ([Yesmin et al., 2020](#_ENREF_308)) |
| *Premna esculenta* Roxb. (Verbenaceae) - Edible Premna (Leloom pata) | **Leaf:** extract is used externally in gout and given orally in hysteria and stomach disorder; paste is applied on snake bite. R**oot:** fresh extract is given in gout, hook worm infestation and ureterolithiasis; pills made from root is given in jaundice, edema and tumor ([Uddin, 2006](#_ENREF_293)) | *In vivo*, carrageenan induced paw edema | Leaf  P.O. 200 mg/kg body wt of each of ethanol extract and its petro-leum ether, carbon tetrachloride, chloroform and ethyl acetate fraction;  Root  P.O. 200 and 400 mg/kg body wt of ethanol extract | Positive control: P.O. diclofenac sodium 50 mg/kg body wt;  Positive control: P.O. diclofenac sodium 50 mg/kg body wt; | *P. esculenta*  extract and fractions inhibited paw edema where significant inhibition obtained with ethyl acetate fraction from 1-4 h after injection with carrageenan;  *P. esculenta* extract inhibited paw edema where maximum inhibition obtained with 400 mg/kg at 2 h after injection with carrageenan | Not determined;  Not determined | ([Qais et al., 2011](#_ENREF_224))  ([Mahmud et al., 2015](#_ENREF_191)) |
| *Ruellia tuberosa* L. (Acanthaceae) - Minnieroot (Patpatey) | **Leaf:** used in the treatment of gonorrhea, ear diseases and stomach cancer; decoction is used in the treatment of bronchitis. **Root:** dried and ground root is used to induce abortion and is used in the treatment of sore eyes  It is also known for its emetic, anthelmintic, diuretic, antipyretic, antidiabetic, antidotal, thirst- quenching, analgesic, antihypertensive and cooling properties and is useful in the treatment of bladder stone, joint pain, strained muscle, urinary problem and uterine fibroids  ([Kader et al., 2014](#_ENREF_152)) | *In vivo,* serotonin and egg albumin induced paw edema | Aerial parts  P.O. 100, 200 and 300 mg/kg body wt of ethanol extract | Positive control: IP indomethacin 5 mg/kg body wt and negative control: P.O. 1% Tween 80 10 mL/kg body wt | 200 and 300 mg/kg of extract significantly inhibited paw edema in 4 h after injection with serotonin as well as egg albumin | Not determined | ([Alam et al., 2009b](#_ENREF_15)) |
| *Sterculia villosa* Roxb. (Sterculiaceae) - Hairy sterculia or elephant rope tree (Udal or udar) | **Leaf:** poultice is applied on the body in hysteria; fresh juice is given in the treatment of lumps in the throat. **Leaf and bark:** paste is applied on forehead to relieve headache. **Leaf and root:** pills prepared from root with honey is given in fever and at the same time leaf juice is used for massaging the whole body; fresh root juice is given orally and at the same time leaf extract is used for hot bath in gout. **Petiole:** infusion is given in calcium deficiency, impotence and obesity. **Bark:** pills prepared from bark are given in jaundice. **Root:** decoction is given in gastric ulcer; fresh juice is given in the treatment of spermatorrhea and spermaturia ([Uddin, 2006](#_ENREF_293)) | *In vivo*, carrageenan induced paw edema and cotton pallet induced granuloma;  *In vitro,* protein (albumin) denaturation and human RBC membrane stabilization assay;  *In vivo*, carrageenan induced paw edema;  *In vitro,* human RBC membrane stabilization assay | Bark  P.O. 100, 200 and 400 mg/kg body wt of ethanol extract;  Leaf  500 μg/mL of ethanol extract;  Seed  P.O. 250 and 500 mg/kg body wt of methanol extract;  Part not found  125, 250, 500, 1,000 μg/mL of ethanol extract | Positive control: P.O. diclofenac sodium 5 mg/kg body wt and negative control: P.O. saline 10 mL/kg body wt;  Positive control: acetyl salicylic acid 1%;  Positive control: P.O. diclofenac sodium 10 mg/kg body wt and negative control: P.O. distilled water 10 mL/kg body wt;  Positive control: diclofenac sodium 125, 250, 500, 1,000 μg/mL and negative control: distilled water | *S. villosa* inhibited paw edema and granuloma formation significantly in a dose dependent manner;  The extract inhibited heat induced protein denaturation as well as hypotonicity induced hemolysis;  The extract inhibited paw edema significantly in a dose dependent manner in the time of 9 h after injection with carrageenan;  1,000 μg/mL of the extract significantly inhibited hypotonicity induced hemolysis; | **Acute toxicity test:** No mortality was observed at a dose of 4,000 mg/kg body wt P.O.;  Not determined;  Not determined;  **Brine shrimp lethality test:** A LC_50_ value of 0.639 μg/mL was obtained with the ethanol extract | ([Hossain et al., 2012b](#_ENREF_128))  ([Tania et al., 2013](#_ENREF_279))  ([Ullah et al., 2015](#_ENREF_296))  ([Anwar et al., 2018](#_ENREF_22)) |
| *Steudnera colocasiifolia* K.Koch (Araceae) - (Bishkachu)  ([Uddin, 2019](#_ENREF_292)) | **Leaf:** paste is used in the treatment of rheumatism ([Uddin, 2019](#_ENREF_292)) | *In vitro,* protein (albumin) denaturation and human RBC membrane stabilization assay | Whole plant 25, 50, 100, 200 and 400 μg/mL of ethanol extract | Positive control: diclofenac sodium 25, 50, 100, 200 and 400 μg/mL | The extract inhibited heat induced protein denaturation as well as hypotonicity induced hemolysis in a dose dependent manner | Not determined | ([Hossain et al., 2015](#_ENREF_130)) |
| *Swietenia mahagoni* (L.) Jacq. (Meliaceae) - American mahogany (Mahagoni) | **Leaf:** decoction is used against nerve ailments. **Leaf and root:** poultices is used to cease bleeding. **Bark:** astringent, antipyretic and tonic; decoction is given in diarrhea and dysentery**;** useful in tuberculosis, anemia, toothache and to increase appetite. **Seed:** decoction is useful in hypertension, controlling blood glucose, constipation, menstrual pain, eczema, rheumatism, improving fertility, fever, cold and to increase appetite; used as insect repellant; infusion is useful against chest pain. **Seed and bark:** aqueous extract is used in psoriasis, diabetes, and diarrhea and also as antiseptic in cuts and wounds ([Sukardiman and Ervina, 2020](#_ENREF_274)) | *In vitro,* human RBC membrane stabilization assay | Bark  1 mg/mL of each of methanol extract and its petroleum ether, carbon tetrachloride and aqueous soluble fraction;  Flower  1 mg/mL of each of methanol extract and its petroleum ether, carbon tetrachloride, chloroform and aqueous soluble fraction | Positive control: acetyl salicylic acid 0.10 mg/mL;  Positive control: acetyl salicylic acid 0.10 mg/mL | 1 mg/mL of extract and fractions inhibited lysis of human RBC membrane induced by hypotonic solution and heat to various extent;  1 mg/mL of extract and fractions inhibited lysis of human RBC membrane induced by hypotonic solution and heat to various extent; | **Brine shrimp lethality test:** LC_50_ values of 0.01, 0.10, 1.15 and 4.70 μg/mL were obtained with methanol extract and its petroleum ether, carbon tetrachloride and aqueous soluble fractions respectively;  **Brine shrimp lethality test:** LC_50_ values of 0.10, 0.45, 1.29, 2.61 and 19.80 μg/mL were obtained with methanol extract and its petroleum ether, carbon tetrachloride, chloroform and aqueous soluble fractions respectively | ([Akter et al., 2014](#_ENREF_10))  ([Rahman et al., 2014](#_ENREF_231)) |
| *Terminalia arjuna* (Roxb. ex DC.) Wight & Arn. (Combretaceae) - Arjuna myrobalan (Arjun gachh)  ([Ghani, 2003](#_ENREF_95)) | **Bark:** as tonic, diuretic, astringent and febrifuge; powdered bark relieves hypertension; it is useful in the treatment of liver cirrhosis, asthma, dysentery, menstrual problems, pain, leucorrhea, wound, skin eruption, bleeding, pus formation in gums, and red and swollen mouth, tongue and gum ([Ghani, 2003](#_ENREF_95)) | *In vivo*, carrageenan induced paw edema;  *In vitro,* human RBC membrane stabilization assay | Stem bark  P.O. 250 and 500 mg/kg body wt of ethanol extract;  Part not found 1 mg/mL of methanol extract | Positive control: P.O. diclofenac 10 mg/kg body wt and negative control: P.O. saline water 10 mL/kg body wt;  Positive control: acetyl salicylic acid 0.10 mg/mL | The extract significantly inhibited paw edema in 8 h after injection with carrageenan;  1mg/mL of methanol extract significantly inhibited lysis of human RBC membrane induced by hypotonic solution and heat | **Acute toxicity test and brine shrimp lethality test:** In acute toxicity test, the extract was administered in graded doses of 50, 100, 250, and 500 mg/kg body wt as single oral dose, observed closely for first 24 h and also next 1 week. No mortality or any significant changes in behavior, breathing, cutaneous effects, sensory nervous system responses or gastrointestinal effects was recorded. On the other hand, in brine shrimp lethality test, a LC_50_ value of 50.11 μg/mL was obtained with the ethanol extract;  Not determined | ([Morshed et al., 2011](#_ENREF_200))  ([Shahriar et al., 2012](#_ENREF_255)) |
| *Terminalia citrina* Roxb. ex Fleming (Combretaceae) - Yellow myrobalan (Haritaki) | **Fruit:** decoction is given in anemia; infusion made from dried fruits is given in cardiac weakness, hyper tension; mixture made from dried fruit with sugar and water is given in constipation; fresh juice is given in dyspepsia; dried fruit powder is given to relieve flatulence, hiccup and is useful in the treatment of edema; pills made with fruit is given is general weakness, hyper acidity, jaundice and respiratory troubles. **Bark:** infusion prepared from dried bark is given in dehydration; juice is useful in spermatorrhea ([Uddin, 2006](#_ENREF_293)) | *In vitro*, human RBC membrane stabilization assay | Leaf  100, 1,000 and 2,000 μg/mL of methanol extract;  100, 1,000 and 2,000 μg/mL of each of methanol extract and its petroleum ether, carbon tetrachloride, dichloro-methane and ethyl acetate fractions | Positive control: naproxen 100, 1,000 and 2,000 μg/mL;  Positive control: naproxen 100, 1,000 and 2,000 μg/mL | *T. citrina* significantly inhibited lysis of human RBC membrane induced by hypotonic in a dose dependent manner;  The extract and its fractions inhibited lysis of human RBC membrane induced by hypotonic in a dose dependent manner | Not determined;  Not determined | ([Das et al., 2015](#_ENREF_69))  ([Das et al., 2016](#_ENREF_70)) |
| *Thunbergia grandiflora* (Roxb. ex Rottl.) Roxb. (Acanthaceae) - Black clock vine, blue trumpet vine  (Kauathuti, nallata or changra morich) | **Leaf:** paste is applied externally in the treatment of gout, hydrocele; pills made from leaf are given in rheumatism; juice is given to cure spermatorrhea. **Bark:** decoction mixed with sugar and honey is given in blood dysentery; pills made from bark with other ingredients are given in marasmus, and pre- and post-eclampsia. **Stem:** sap obtained from cutting the stem is applied into the eyes in ophthalmia, cataract and conjunctivitis; pills made from dried stem are given in diabetes. **Root:** fresh juice is given to cure hysteria and in stomachache. **Whole plant:** decoction mixed with honey is given to cure malaria ([Uddin, 2006](#_ENREF_293)) | *In vitro*, human RBC membrane stabilization and protein (albumin) denaturation assay | Leaf  31.25, 62.5, 125, 250,  500 and 1,000 μg/mL of methanol extract | Positive control: diclofenac sodium 31.25, 62.5, 125, 250, 500 and 1,000 μg/mL | Different concentration of methanol extract significantly inhibited hemolysis induced by hypotonic solution as well as protein denaturation in a dose dependent manner | Not determined | ([Alam et al., 2015](#_ENREF_16)) |
| *Toona ciliata* M.Roem*.* (Meliaceae) - Red toon, red cedar, or Indian mahogany  (Piya tun, peo nandibriksha or rangi)  ([Ghani, 2003](#_ENREF_95)) | **Flower:** useful in menstrual disorders. **Bark:** astringent, tonic and antiperiodic; infusion is given in chronic dysentery, fever and rheumatism; powdered bark is applied to ulcers ([Ghani, 2003](#_ENREF_95)) | *In vivo*, carrageenan and histamine induced paw edema | Leaf  P.O. 200 and 400 mg/kg body wt of ethanol extract | Positive control: P.O. indomethacin 10 mg/kg body wt and negative control: P.O. 1 % Tween 80 in normal  saline 10 mL/kg body wt | 400 mg/kg body wt of *T. ciliata* extract significantly inhibited paw edema in 4 h after injection with carrageenan as well as with histamine | Not determined | ([Hossain et al., 2014a](#_ENREF_125)) |
| *Typhonium trilobatum* (L.) Schott (Araceae) - Bengal arum (Ghatkanchu, ghatkol) | **Tuber and root:** stimulant; useful in the treatment of piles and as poultice to tumor; given with bananas to cure stomach complaints; applied externally and given internally at the same time in case of snake bite ([Ghani, 2003](#_ENREF_95)) | *In vivo*, xylene induced ear edema;  *In vitro,* human RBC membrane stabilization assay;  *In vitro,* protein (albumin) denaturation and human RBC membrane stabilization assay | Leaf  250, 500 mg/kg body wt of ethanol extract;  Whole plant  1 mg/mL of successive methanol, ethanol and chloroform extract;  Leaf  1 mg/mL of methanol extract and its chloroform and ethanol fraction;  Root  0.01, 0.02, 0.04, 0.06 and 0.08% (v/v) of aqueous extract | Positive control: P.O. diclofenac sodium 10 mg/kg and negative control: 1% Tween 80 in water 10 mL/kg body wt;  Positive control: acetyl salicylic acid 0.1 mg/mL;  Positive control: acetyl salicylic acid 0.1 mg/mL;  Positive control: acetyl salicylic acid 100 μg/mL | 250 and 500 mg/kg of extract significantly inhibited xylene induced ear edema in a dose dependent manner;  *T.* *trilobatum* extracts inhibited both heat and hypotonicity induced hemolysis;  1 mg/mL of crude methanol extract and its fractions inhibited both heat and hypotonicity induced hemolysis;  The extract inhibited heat induced protein denaturation as well as hypotonicity induced hemolysis in a dose dependent manner; | **Acute toxicity test:** The extract was administered in graded doses with a maximum dose of 800 mg/kg body wt IP. The LD_50_ was claimed to be more than 800 mg/kg body wt since no mortality or toxicity was recorded up to this dose during the observation period of 48 h;  **Acute toxicity test:** The extracts were administered in graded doses of 100, 500 and 1,000 mg/kg body wt as single oral dose, observed closely for first 5-6 h and also next 2 weeks. No mortality or toxicity was noted;  **Acute toxicity test:** The extract and fractions were administered as a single oral dose of 1,000 mg/kg body wt, observed closely for 24 h and also for next 1 week. No mortality or toxicity was noted;  ***Allium cepa* toxicity test:** Allium cepa test was conducted with 2.5, 5, 10, 15 and 20% (v/v) concentration of the aqueous extract for 24, 48 and 72 h. Lowest root growth inhibition was observed at exposure time of 48 h in 2.5% (v/v) concentration, while highest at 72 h in 20% (v/v) concentration. Therefore the result indicates that the extract exerted toxic effects at high concentrations. | ([Ali et al., 2012](#_ENREF_17))  ([Shahriar et al., 2015](#_ENREF_256))  ([Shafa et al., 2016](#_ENREF_252))  ([Parvin et al., 2019](#_ENREF_219)) |
| *Urena sinuata* L. (Malvaceae) - Bur mallow (Atapuran)  ([Uddin, 2019](#_ENREF_292)) | **Leaf and root:** decoction is used to soften the skin  Other medicinal uses of this plant includes treatment of sprain, bruises, skin disease, gonorrhea, fever, malaria, pain and inflammation ([Uddin, 2019](#_ENREF_292)) | *In vivo*, carrageenan induced paw edema and cotton pallet induced granuloma | Leaf  P.O. 200 and 400 mg/kg body wt of chloroform extract | Positive control: P.O. diclofenac sodium 10 mg/kg body wt and negative control: P.O. 1% v/v Tween-80 in water 10 mL/kg body wt | 400 mg/kg of *U. sinuata* extract significantly inhibited paw edema as well as granuloma formation | **Acute toxicity test:** Extract was administered in graded doses of 100, 200, 300, 600, 800, 1,000, 2,000 and 3,000 mg/kg body wt as single oral dose, observed closely for first 8 h and also for next 2 weeks. No mortality or behavioral changes like sedation, excitability etc. or allergic manifestations was noted. | ([Emran et al., 2018](#_ENREF_80)) |
| *Vigna unguiculata* (L.) Walp. (Fabaceae) - Cowpeaor Chinese bean (Barbati)  ([Uddin, 2019](#_ENREF_292)) | **Seeds:** are sweet and have laxative, astringent, anthelmintic, anti-bacterial, diuretic and galactagogue properties; useful in the treatment of inflammation, jaundice, anorexia and general disability  ([Hussain et al., 2019](#_ENREF_135)) | *In vitro,* human RBC membrane stabilization assay and *in vivo,* carrageenan induced paw edema | Seed  For *in vitro* assay, 1, 3, 5, 7 and 9 mg/mL of methanol extract and for *in vivo* assay, P.O. 200 and 400 mg/kg body wt of methanol extract | For *in vitro* assay, positive control: acetyl salicylic acid 0.1 mg/mL and for *in vivo* assay, positive control: P.O. ibuprofen 10 mg/kg body wt | Different concentration of methanol extract inhibited hemolysis induced by hypotonic solution and heat in a dose dependent manner and 400 mg/kg of *V.* *unguiculata* extract significantly inhibited paw edema in 4 h after injection with carrageenan | Not determined | ([Hussain et al., 2019](#_ENREF_135)) |
| *Vitex negundo* L. (Verbenaceae) - Chaste tree  (Nishinda or samalu)  ([Ghani, 2003](#_ENREF_95)) | **Leaf:**  antiparasitic, vermifuge, alterative and anodyne and useful against rheumatic attack. **Flower:** astringent and cooling agent; oil from flower is applied to sinus infection and scrofulous sores. **Fruit:** nerve tonic, emmenagogue and vermifuge**. Root:** tonic, febrifuge, expectorant, diuretic, breast-milk enhancer and progestogenic  ([Ghani, 2003](#_ENREF_95)) | *In vitro*, rat RBC membrane stabilization assay;  *In vitro*, human RBC membrane stabilization assay | Leaf  0.25, 0.5, 1.0, 1.5 and 2.0 mg/mL of ethanol extract;  Bark  1.0 mg/mL of each of methanol extract and its *n*-hexane, carbon tetrachloride, chloroform and aqueous soluble fraction | Positive control: indomethacin 0.1 mg/mL;  Positive control: acetyl salicylic acid 0.1 mg/mL | Different concentration of methanol extract significantly inhibited hemolysis induced by hypotonic solution in a dose dependent manner;  1 mg/mL of aqueous soluble fraction significantly protected the lysis of human erythrocyte membrane induced by hypotonic solution | Not determined;  **Brine shrimp lethality test:** LC_50_ values of 102.62, 3.71, 130.97, 0.91, and 39.78 μg/mL were obtained with methanol extract and its *n*-hexane, carbon tetrachloride, chloroform and aqueous soluble fractions respectively | ([Alam et al., 2009a](#_ENREF_14))  ([Khan et al., 2013](#_ENREF_163)) |
| *Withania somnifera* (L.) Dunal (Solanaceae) - Winter cherry (Ashwagondha or Dhuppa)  ([Ghani, 2003](#_ENREF_95)) | **Leaf:** sedative. **Leaf and root:** alterative, tonic, diuretic, deobstruent, astringent, aphrodisiac and sedative; both are used in the treatment of headache, convulsion, insomnia, hiccup, cough, rheumatism, dropsy, seminal weakness and mouth sore; bruised leaves and ground roots are applied locally to treat painful swelling, carbuncles and ulcers. **Root:** efficacious in general debility, rheumatism and memory loss. **Fruit and seed:**  diuretic and hypotonic ([Ghani, 2003](#_ENREF_95)) | *In vitro*, human RBC membrane stabilization assay | Part not found 1 mg/mL of methanol extract;  Root  1 mg/mL of each of methanol, ethanol and chloroform extract | Positive control: acetyl salicylic acid 0.10 mg/mL;  Positive control: acetyl salicylic acid 0.10 mg/mL | 1mg/mL of methanol extract significantly inhibited lysis of human RBC membrane induced by hypotonic solution and heat;  1 mg/mL of each of methanol, ethanol and chloroform extract inhibited lysis of human RBC membrane induced by hypotonic solution and heat where maximum inhibition exerted by the methanol extract | Not determined;  Not determined | ([Shahriar et al., 2012](#_ENREF_255))  ([Shahriar et al., 2014](#_ENREF_254)) |
